# Using light scattering to assess how phospholipid–protein interactions affect complex I functionality in liposomes†

**DOI:** 10.1039/d2cb00158f

**Published:** 2023-03-20

**Authors:** Jana Eisermann, John J. Wright, James D. E. T. Wilton-Ely, Judy Hirst, Maxie M. Roessler

**Affiliations:** a Department of Chemistry, Imperial College London, Molecular Sciences Research Hub, White City Campus London W12 0BZ UK m.roessler@imperial.ac.uk j.wilton-ely@imperial.ac.uk; b The Medical Research Council Mitochondrial Biology Unit, University of Cambridge, The Keith Peters Building, Cambridge Biomedical Campus Cambridge CB2 0XY UK

## Abstract

Complex I is an essential membrane protein in respiration, oxidising NADH and reducing ubiquinone to contribute to the proton-motive force that powers ATP synthesis. Liposomes provide an attractive platform to investigate complex I in a phospholipid membrane with the native hydrophobic ubiquinone substrate and proton transport across the membrane, but without convoluting contributions from other proteins present in the native mitochondrial inner membrane. Here, we use dynamic and electrophoretic light scattering techniques (DLS and ELS) to show how physical parameters, in particular the zeta potential (*ζ*-potential), correlate strongly with the biochemical functionality of complex I-containing proteoliposomes. We find that cardiolipin plays a crucial role in the reconstitution and functioning of complex I and that, as a highly charged lipid, it acts as a sensitive reporter on the biochemical competence of proteoliposomes in ELS measurements. We show that the change in *ζ*-potential between liposomes and proteoliposomes correlates linearly with protein retention and catalytic oxidoreduction activity of complex I. These correlations are dependent on the presence of cardiolipin, but are otherwise independent of the liposome lipid composition. Moreover, changes in the *ζ*-potential are sensitive to the proton motive force established upon proton pumping by complex I, thereby constituting a complementary technique to established biochemical assays. ELS measurements may thus serve as a more widely useful tool to investigate membrane proteins in lipid systems, especially those that contain charged lipids.

## Abstract 2

Complex I is an essential membrane protein in respiration and contributes to ATP synthesis. Zeta potential measurements are shown to be sensitive to the enzyme’s activity and capability to pump protons in varying lipid environments.

## Graphical abstract

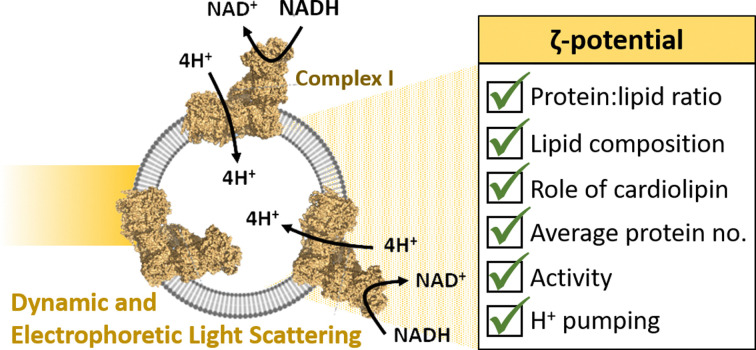

## Introduction

All living cells contain lipid-based membranes that separate them from their external environments and allow intracellular compartmentalization. Membranes are formed from bilayers of amphiphilic phospholipid molecules. In biochemical systems, they harbour small hydrophobic components, such as vitamins, quinones and pigments, in addition to a huge range of membrane proteins from small, peripherally bound proteins to integral multicomponent megadalton complexes.^1^ The total protein content of a membrane increases with the complexity of the biochemical functions it helps to sustain, with the mass percentage of protein varying between 25% in the myelin sheath and 75% in the mitochondrial inner membrane.^2^ Moreover, the structure and composition of the lipid molecules that constitute the membrane modulate the structures and activities of proteins by affecting both inter- and intra-molecular interactions.^3^

The innate complexity of densely packed biological membranes makes targeted functional studies of membrane proteins in their native cellular environment challenging. To overcome the problem, mimetic systems such as membrane-monolayers, protein wrapped lipid patches (nanodiscs) as well as polymer-encapsulated lipid particles, planar lipid bilayers, lipid vesicles (known as liposomes)^1^ and hybrid vesicles^4^ have been employed. These systems enable membrane proteins to be studied in relatively simple, well-defined environments while still being orientated and inserted inside a lipid bilayer system for their full activity,^5^ a feature that is especially relevant for proteins with vectorial transport functions. Due to their versatility, liposomes have become the workhorses for studying membrane proteins in a near-native environment.^6^ Liposomes are typically spherical structures and can be formed *in vitro* with controlled lipid compositions and with discrete sizes by choosing a suitable preparation procedure (*e.g.* sonication, extrusion, reverse-phase evaporation or electroformation). They are classed according to their diameter: small unilamellar vesicles (SUVs, 30 nm to 100 nm), large unilamellar vesicles (LUVs, 100 nm to 1 μm) and giant unilamellar vesicles (GUVs, up to 50 μm).^1,6^ Choosing the right diameter can be crucial and depends on the intended application of the vesicles and the techniques used for their characterisation.

One of the most intriguing membrane proteins in mitochondria and prokaryotic cells is respiratory complex I (NADH:quinone oxidoreductase, henceforth **R-CI**). R-CI is crucial for cellular metabolism in humans and other aerobic organisms and plays a major role in ATP synthesis. R-CI oxidises NADH (primarily from the tricarboxylic cycle and β-oxidation) at a flavin mononucleotide (FMN) with the two electrons transferred sequentially down a long chain of seven iron–sulfur clusters to ubiquinone (Q_10_), and the redox potential difference used to pump four protons across the mitochondrial inner membrane.^7,8^ R-CI thus contributes substantially to the proton motive force that is used to drive ATP synthesis. While NADH oxidation and electron transfer through the iron–sulfur clusters are relatively well understood, the mechanism of Q_10_ reduction remains unresolved,^9^ not least due to the hydrophobicity of Q_10_ itself,^10^ which renders studies with the native substrate in isolated systems difficult.^11–14^

To be able to use Q_10_ whilst studying R-CI separately from the other proteins present in the mitochondrial inner membrane, R-CI has been incorporated into liposomes (LUVs) to form proteoliposomes (PLs). Detergent-mediated reconstitution has been used with R-CI isolated from bacteria,^15–18^ fungi (*Yarrowia lipolytica*^9,19,20^ and *Pichia pastoris*^9^) and mammalian cells (*Bos taurus*^9,14,20–22^). Artificial membrane environments can be constructed with varying complexity, ranging from simple soybean extracts such as asolectin, to bovine heart lipid extracts, purified natural lipids and synthetic phospholipid mixtures. The latter have the advantage that the influence of every component of the liposome membrane can be characterised. Moreover, they fulfil the attributes for a ‘good’ lipid mixture: facile liposome formation, preservation of enzyme activity and resistance of the liposome towards protons or other ions.^1^ Indeed, the activity of R-CI with the native Q_10_ substrate could be determined with such well-defined liposomes, where the additional incorporation of the alternative oxidase (AOX) was used to re-oxidise the quinone pool inside the PL membrane without interfering with proton translocation across the membrane.^14,22^

Despite these R-CI PL studies, the morphology of PLs has not been investigated comprehensively, and there is very limited understanding of the relationship between lipid composition and enzyme activity. For R-CI, the main lipid components of the native membrane are phosphatidylcholine (PC), phosphatidylethanolamine (PE), and cardiolipin (**CL**).^23^ CL is of particular importance as a unique tetra-acyl anionic phospholipid that is essential for the function and/or structural integrity of several complexes in the mitochondrial inner membrane, where it is mainly localised.^24^ CL plays a major role in many mitochondrial processes (*e.g.* respiration and energy conversion) and pathological changes in the amount and fatty-acid composition of CL can have negative consequences for mitochondrial functionality.^25^ Loss of CL was found in numerous studies to result in dysfunctional oxidative phosphorylation machinery, altered mitochondrial morphology and/or the elevated production of reactive oxygen species.^26^ The functional importance of CL seems to be linked to its unique ability to interact with proteins and in its role to maintain both inner membrane fluidity as well as osmotic stability.^25^ Furthermore, CL deficiency may impede ATP synthesis due to impaired respiratory chain function.^27^ CL binds at specific sites on the R-CI membrane domain, mainly driven by electrostatic interactions between the lipid headgroup and positively charged protein residues. This binding further modulates intersubunit contacts, which are thought to play an important role in proton transport across the membrane domain.^28^

In this study, we develop a systematic approach that combines dynamic and electrophoretic light scattering (DLS, ELS) measurements with established biochemical assays,^14,22^ to correlate the lipid composition of the liposome membrane with its ability to incorporate and support functional R-CI. In particular, the role of CL in the formation of functional PLs and its association with R-CI is investigated. We show that the zeta potential (*ζ*-potential), also termed the electrokinetic potential,^29^ provides an excellent guide to the quantity and quality of reconstituted R-CI in PLs, and is responsive to the membrane potential established as a result of the enzyme's proton pumping activity.

## Materials and methods

All main parameters used in our study can be found in Table S1 (ESI†).

### Chemicals for Q_10_-loaded liposomes

Synthetic lipids DOPC (1,2-dioleoyl-*sn*-glycero-3-phospho-choline), DOPE (1,2-dioleoyl-*sn*-glycero-3-phosphoethanol-amine) and 18:1-cardiolipin were purchased as chloroform stock solutions with a concentration of 10 or 25 mg mL^−1^ (Avanti Polar Lipids). Q_10_ (Sigma-Aldrich) was dissolved in chloroform (VWR Chemicals) to a stock concentration of 10 mM. For the reconstitution buffer, 10 mM of MOPS (Sigma Life Science) and 50 mM KCl (Sigma-Aldrich) were dissolved in Millipore MilliQ water (specific resistance *ρ* = 18.2 MΩ⋅cm), adjusted to pH 7.4 with concentrated NaOH. All chemicals were used without further purification.

### Protein purification

Overexpression and purification of AOX from *Trypanosoma brucei brucei*^30^ and preparation of R-CI from *Bos taurus* heart mitochondria^30^ were performed as described previously, concentrated to 15–25 mg mL^−1^ (for R-CI) and 5–8 mg mL^−1^ (for AOX) and stored at −80 °C. Bicinchoninic acid (BCA) assays were used to determine the concentrations of purified enzymes.^31^

### Preparation of liposomes

Liposomes (LUVs) were prepared using thin-film rehydration of phospholipid mixtures and extrusion.^32^ Synthetic lipids (10 mg from chloroform stock solutions) at a mass ratio of 8 : 1 : 1 DOPC : DOPE : CL were mixed with 100 nmol Q_10_ in a 7 mL glass vial, to generate the ‘optimal composition’ referred to throughout.^22^ Variations in the lipid composition are specified in the figure captions, legends and Table S2 (ESI†). Chloroform was removed using a gentle stream of N_2_(g), creating a thin film of lipids at the bottom of the vial. To remove residual solvent, the vial was placed in a vacuum desiccator for 1 h. The dried film was then rehydrated for at least 30 min by adding 1 mL of reconstitution buffer, during which the lipid–Q_10_ mixture was vortexed periodically. The 10 mg mL^−1^ lipid solution was then extruded using a hand-held Mini-Extruder (Avanti) equipped with a 100 nm polycarbonate membrane (Whatman). 31 passes through the membrane were performed to guarantee monodisperse size distributions^33^ as determined by DLS measurements (extrusion I).

For lipid concentration measurements a second extrusion was performed. The Mini Extruder setup was not disassembled after the first extrusion, but the syringe was loaded with a defined volume of reconstitution buffer. The buffer solution was passed 31 times (extrusion II) through the same membrane to collect the remaining lipids in the extruder. The loaded and collected sample volumes from both extrusions were then recorded.

### Preparation of PLs

The preparation of PLs was adapted from a previously reported protocol.^22^ Briefly, 250 μL of Q_10_-containing liposomes (lipid content 10 mg mL^−1^) were partially solubilised with sodium cholate from a 20% (w/v) stock (final detergent concentration 0.6%). The lipid-detergent mixture was unified by inversion and incubated on ice for 10 min. R-CI was added in a protein : lipid (w/w) ratio of 1 : 12.5, 1 : 25, 1 : 50 or 1 : 100 (as specified), with gentle shaking of the solution. After 15 min incubation on ice, the solution was passed through a PD10 desalting column (Cytiva) at 4 °C to remove the detergent. The eluate containing the PLs was centrifuged at 120 000 × *g* (4 °C, 2.5 h) using a fixed-angle 45Ti rotor (Beckman Coulter). To enable use of the 70 mL polycarbonate tubes required for the rotor, the sample was diluted with 30 mL buffer solution. The pellet was resuspended in 200 μL ice-cold reconstitution buffer and stored on ice until use.

The successful preparation of bovine R-CI PLs with optimal composition was ascertained and compared with literature values where possible (Table S3, ESI†). Different batches of purified bovine R-CI affected the overall performance of the PLs prepared with the same reconstitution procedure. However, we observed similar changes in both physical and biochemical parameters.

For the proton pumping and biochemical characterisation experiments, AOX was added to diluted PL solutions in variable amounts (0–10 μg mL^−1^) following reconstitution with R-CI.^30^

### Physical characterisation of liposomes and PLs

All light scattering measurements were performed with PLs containing R-CI and Q_10_ only, unless the addition of AOX is explicitly stated. R-CI catalysis was initiated by adding NADH to PLs containing R-CI, AOX and Q_10_.

#### Dynamic light scattering (DLS)

Size distributions were determined using a Zetasizer Ultra instrument (Malvern Instruments Ltd) at 25 °C and a small-volume quartz cuvette (Hellma) containing 60 μL of the solution to be investigated. Before the measurement, vesicle stock solutions were diluted at least 10-fold with reconstitution buffer to prevent multiple scattering effects.^34,35^

To improve the resolution limit of simple single angle DLS, all measurements were performed using Multi-Angle Dynamic Light Scattering (MADLS, see also Section 3.1 in the ESI†).^35,36^ The additional time required *vs.* “standard” DLS was minimal (∼5 min per MADLS *vs.* ∼2 min per DLS measurement, including three replicates). The intensity-weighted distributions were calculated with the instrument-specific software ZS Xplorer (version 1.3). Three replicates from one PL batch were prepared, with each MADLS measurement performed in triplicate (*i.e.* 9 measurements in total) to ensure reproducibility. The settings for the dispersant were viscosity *η* = 0.887 mPa s and refractive index *n* = 1.33.

#### Electrophoretic light scattering (ELS)

ELS measurements were performed in folded capillary cells (Malvern Instruments Ltd) using a Zetasizer Ultra instrument with ZS Xplorer software (using Phase Analysis Light Scattering, PALS), allowing evaluation of the *ζ*-potential for sensitive samples (*e.g.* highly conductive protein solutions).^29,37^ The diffusion barrier technique enabled the use of low sample volumes (60 μL) and high ionic strength buffers: the sample (at least 10-fold diluted stock vesicle solution, as for DLS measurements) was injected into the bottom of the cell containing the reconstitution buffer and the applied voltage was limited to 80 V to prevent Joule heating^38^ inside the cuvette, which could lead to protein degradation. The measured electrophoretic mobilities were averaged, and *ζ*-potentials were calculated as described in Section 3.4 (and shown in Table S4, ESI†) of the ESI.† The settings for the solvent were viscosity *η* = 0.887 mPa s, refractive index *n* = 1.33, and dielectric permittivity *ε*_r_ = 78.5.

To measure proton pumping and the build-up/collapse of the proton motive force, defined volumes of either NADH (final concentration 200 μM) and then valinomycin (final concentration 0.1 μM) were injected into the sample area at the bottom of the folded capillary cell. After the injection, ELS measurements were repeated five times under the same conditions to ensure that no degradation occurred as a result of the applied alternating electric field. Including the 90 s break between each ∼30 s long ELS measurement, each sample thus took ∼10 min to record. Three replicates from one PL batch were prepared for each ELS sample.

### Lipid concentration determination for liposomes and PLs (*c*_lipid_)

The total lipid concentration for both liposomes and PLs with increasing CL content (0–10 wt%) was determined using the two approaches below, based on Di Prima *et al.*^39^ The total phospholipid content was 7.3–8.6 mg mL^−1^ for all lipid compositions.

#### Stewart assay

The formation of a coloured complex between the phospholipids (dissolved in chloroform) and the assay solution of 0.1 mM ammonium ferrothiocyanate was used to determine the lipid content. The assay solution was prepared by dissolving 27.03 g L^−1^ of FeCl_3_ (VWR Chemicals) and 30.40 g L^−1^ of NH_4_SCN (Fluorochem) in Milli-Q water.^40^ To generate the calibration curve, lipid stock solutions were diluted to 0.005–0.05 mg mL^−1^ with chloroform. Since the lipid composition affects the calibration curve, each lipid mixture of interest was measured. To determine the lipid concentration in liposomes and PLs, 5–10 μL of each vesicle solution was added to 2 mL of chloroform, then vortexed and left for 1 h to extract the lipids. The lipid-containing chloroform solution (2 mL) was vortexed with the assay solution (2 mL) for 30–60 s and left for 1–2 h to separate the aqueous and organic layers. The organic layer was extracted and the absorbance at 485 nm was measured with a Shimadzu 1910i UV-Vis spectrometer. Triplicates for each liposome/PL sample were prepared and measured.

#### Light scattering

The weight concentration of the lipids was obtained by analysing the static light scattering intensity (stated as mean derived count rate in the ZS Xplorer software), measured at side and back scattering (90° and 173°). Since the intensity is linked to *e.g.* the form factor of vesicles (see ESI† Section 3.3), two samples with the same form factor (as shown by similar size distributions with MADLS) were compared to obtain the lipid concentration. For liposomes, extrusion I and II were used, whereas the PLs were correlated to the extrusion I sample. The MADLS measurements were performed with samples diluted in reconstitution buffer (1 : 500 for extrusion I, 1 : 50 for extrusion II, 1 : 200 for PLs). The following parameters, obtained from the MADLS measurements (see ESI† Section 3.3) and complementary spectrofluorometric measurements (see below), contributed to the calculations used to determine lipid concentrations: the scattered light intensity (*I*_1_ and *I*_2_ from extrusions I and II, respectively) obtained after subtracting the value for just the reconstitution buffer, sample dilution (*d*_1_ and *d*_2_), extruded volume (*V*_1_ and *V*_2_), starting total mass of lipids (*M*_0_) and unrecovered/dead volume (Δ*V*).

The spectrofluorometric measurements were performed with an Agilent Cary Eclipse instrument (Agilent Technologies). The MADLS samples were used, and the elastic peak of the vesicle dispersions were recorded by setting equal values for the excitation and emission wavelength (here 532 nm). The excitation and emission slit widths were set to 5 nm. The area under the elastic peak was determined and applied in the same calculations as for the DLS (the area values represent *I*_1_ and *I*_2_). Triplicates for each liposome/PL sample were prepared and measured.

### Biochemical characterisation of PLs

All spectrophotometric kinetic assays were carried out in reconstitution buffer (10 mM MOPS, pH 7.4, 50 mM KCl) at 32 °C using a SpectraMax M3 96 well plate reader (Molecular Devices) and Falcon 96 well clear microplate. For the fluorescence-based assays, the Agilent Cary Eclipse spectrofluorometer was used in combination with a standard quartz cuvette (Hellma) at 32 °C.

#### NADH:APAD^+^

R-CI content and orientation in PLs were determined using the NADH:APAD^+^ oxidoreduction assay, which reports on the activity of the flavin (NADH oxidation) site of complex I in the presence of 100 μM NADH (Alfa Aesar), 500 μM APAD^+^ (Sigma-Aldrich) and 1 μM piericidin A (Sigma-Aldrich).^14^ Note that lipid composition does not affect flavin-site activity,^41,42^ but rather the efficiency of incorporating R-CI into liposomes. To assess the orientation of R-CI, the assay was conducted in the presence/absence of the pore-forming antibiotic alamethicin (20 μg mL^−1^, Cayman Chemical). Absorbance was measured at 400 nm and 450 nm (*ε*_400–450_ = 3.16 mM^−1^ cm^−1^).

#### NADH:O_2_

The ‘overall’ catalytic activity of reconstituted R-CI (*i.e.* including quinone turnover and therefore catalytic rates) was determined from the NADH:O_2_ oxidoreduction rate, monitored at 340 nm and 380 nm (*ε*_340–380_ = 4.81 mM^−1^ cm^−1^) and using 200 μM NADH, 10 μg mL^−1^ AOX and 1.5 μg mL^−1^ outward-facing protein concentration.^22^

#### ACMA fluorescence quenching assays

ACMA (9-amino-6-chloro-2-methoxyacridine) assays to assess proton pumping were performed by transferring PLs to the reconstitution buffer containing 0.5 μM ACMA and 0.1 μM valinomycin (both Sigma-Aldrich). For NADH:O_2_ ACMA assays, AOX was added at 1 μg mL^−1^ (unless otherwise stated) and proton pumping initiated by addition of 500 μM NADH. Dissipation of the proton gradient was induced by the addition of 10 μg mL^−1^ alamethicin. Since constant stirring was not possible with the fluorimeter used, the measurement was paused before each injection to allow manual mixing of the sample. Due to these pauses (∼25 s), there are two short sections with no data points in the ACMA traces.

### Calculations based on physical and biochemical parameters

Both physical (*e.g.* size, *ζ*-potential) and biochemical (*e.g.* protein retention, orientation, activity) analyses were used to characterise the PLs further.

#### Number of vesicles *N*_ves_

MADLS measurements (performed with the Zetasizer) allowed determination of the concentration of dissolved vesicles.^35^ The correlation between the intensity-weighted size-distribution and the number of vesicles per unit volume then leads to *N*_ves_ and is described in the ESI† (Section 3.2). For the ZS Xplorer software to perform the calculation that gives *N*_ves_, the scattering intensity from each sample must be corrected for solvent contributions. The detected photon count rate of the instrument was normalised based on the instrument detection efficiency, which was determined with a toluene reference sample.

#### Protein concentration *c*_R-CI_

To determine the concentration of reconstituted R-CI in PLs, the rate of NADH:APAD^+^ oxidoreduction with alamethicin was compared to the rate from detergent-solubilized R-CI (10 μg mL^−1^ R-CI in reconstitution buffer with 100 μM NADH, 500 μM APAD^+^, 500 nM piericidin A, and 0.2% (w/v) DDM (Sigma-Aldrich) where applicable).

#### Average number of R-CI per PLs

Based on *c*_R-CI_, the total number of R-CI molecules (*N*_R-CI_) can be obtained, taking into account the sample volume, Avogadro's number and the molecular weight of R-CI (see ESI† Section 4.1). The average R-CI number per PL was then obtained by dividing *N*_R-CI_ by the measured number of vesicles.

#### Number of CL molecules per PLs

The lipid concentration per vesicle was calculated by dividing the total lipid concentration (*c*_lipid_) by the number of vesicles (*N*_ves_). To then determine the concentration of CL, and hence the number of CL molecules (see ESI† Section 4.1 with Table S5, ESI†), the weight percentage value in PLs was assumed to remain unchanged in the reconstitution process. The ratio of the vesicle to CL concentration then provided the number of CL per PL.

## Results

### Parameters used to characterise liposomes and PLs

In this work, we use physico-chemical methods to complement biochemical approaches to understand how phospholipid–protein interactions impact on R-CI function. We thus briefly discuss the physical nature of charged proteoliposome vesicles^38,43,44^ and the biochemical assays employed (see also Table S1 in the ESI† for a parameter overview).

**Fig. 1:**
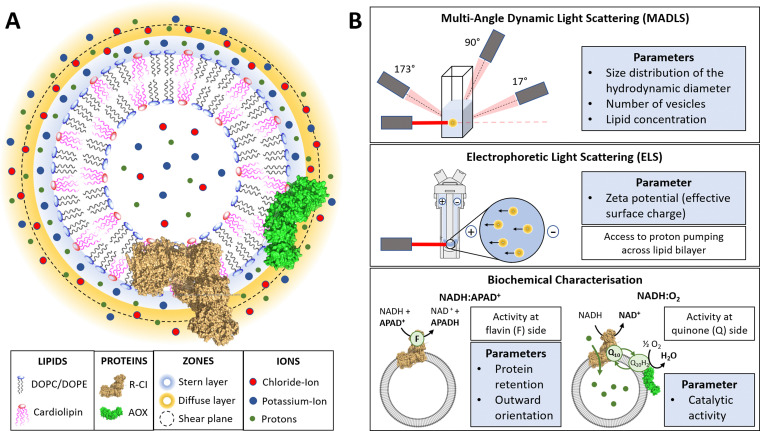
Introduction to PLs and characterisation parameters. (A) Schematic representation of PLs containing R-CI (PDB 5LDW) as well as AOX (PDB 3VV9) which was added for all activity assays focusing on the Q_10_ site to re-oxidise the quinone pool. The lipid bilayer contains the synthetic lipids DOPC, DOPE and 18 : 1 CL in a defined ratio. Immediately surrounding the liposome is a layer of tightly associated ions, opposite in charge to the surface of the liposome (here potassium ions). Surrounding this Stern layer is a second layer (diffuse layer) of loosely associated ions. The point at which the second layer of ions moves with the liposome as a single entity is termed the slipping plane. This plane defines the *ζ*-potential. (B) Summary of the main physico-chemical and biochemical characterisation techniques.

The liquid surrounding a vesicle can be divided into an inner region (Stern layer) dominated by strongly bound ions with opposite charges to the surface, and an outer region (diffuse layer) with more loosely associated ions at concentrations closer to the electroneutral bulk values. Due to the presence of an anionic phospholipid in our vesicle systems (CL), positively charged potassium ions from the reconstitution buffer build up the Stern layer (see Fig. 1A).^45^

A hypothetical boundary exists within the diffuse layer, inside of which the vesicle and associated ions are considered a stable/rigid entity. Thus, under the influence of an external electric field, the ions inside this boundary move with the moving vesicle, whereas ions outside the boundary migrate independently in the dispersion medium (Fig. 1A). The ***ζ*****-potential**, measured *via* electrophoretic light scattering (ELS, Fig. 1B), describes the electrostatic potential at this imaginary boundary or ‘slipping plane’. The *ζ*-potential is thus insensitive to the composition of the liposome lumen. The slipping plane is also the surface at which vesicles are considered to interact, and the **hydrodynamic diameter** (*d*_H_) represents the size of the vesicle up to the slipping plane.^46^ The hydrodynamic diameter, accessible *via* dynamic light scattering (**DLS**), therefore differs from the physical vesicle diameter.

While measuring the *ζ*-potential to characterise the surfaces of charged vesicles is simple, data analysis can be complex due to phenomena such as the position of the slipping plane or orientation of the lipid headgroups.^47^ Thus, we primarily discuss relative changes in the *ζ*-potential. The interdependence of the parameters (*e.g.* knowing the vesicle hydrodynamic diameter is a prerequisite for determining the *ζ*-potential) and workflow for determining the *ζ*-potential is summarized in ESI† Section 3.4 and Scheme S1 (ESI†).

Using a multi-angle approach (**MADLS**, Fig. 1B) when carrying out DLS measurements delivers angular-independent size distributions with improved resolution. The more detailed insight into all size populations present in a given sample further informs on the total **number of vesicles (*N*_ves_)**, *i.e.* the number of liposomes or PLs per unit volume (mL) of sample.^48^ In *N*_ves_ measurements, the time-averaged photon count-rate scattered by the sample is recorded and then transformed into a vesicle distribution (see ESI† Section 3.2). The total **lipid concentration** (***c***_**Lipid**_) in vesicles was derived from both DLS measurements and the Stewart assay (see Methods)^39^ and, in combination with *N*_ves_, allowed calculation of the average number of phospholipids and CLs per vesicle.

The **NADH:APAD**^**+**^ assay^14^ characterises the total R-CI **flavin-site activity**. Although the lipid composition does not affect R-CI flavin-site activity intrinsically,^41,42^ it affects the efficiency of incorporating R-CI (termed **protein retention**) and hence the observed flavin-site activity. The amount of **outward-facing** R-CI can be determined by adding a pore-forming agent such as alamethicin, which makes the membrane permeable to NADH/APAD^+^. This yields the **total R-CI concentration** (***c***_**R-CI**_), which is compared with the number of R-CI whose hydrophilic domain faces outwards. The average number of R-CI per PL can then be determined from *N*_ves_ and *c*_R-CI_. Given that the lipid composition is known, the CL:protein ratio in PLs can also be determined. The **catalytic activity** of reconstituted R-CI at the quinone site was obtained from the **NADH:O**_**2**_ oxidoreduction rate. Sustained R-CI turnover and hence the build-up of the **proton motive force (PMF)** requires the presence of AOX and quinone (Q_10_) in the PLs. The PMF itself is comprised of two components, the membrane potential **Δ*****ψ*** (*i.e.* difference in charge) and the proton concentration gradient **ΔpH**. **ACMA quenching assays** measure ΔpH since Δ*ψ* is dissipated throughout the experiment by an ionophore.

### *ζ*-Potential sensitivity to lipid composition and PL integrity

**Fig. 2:**
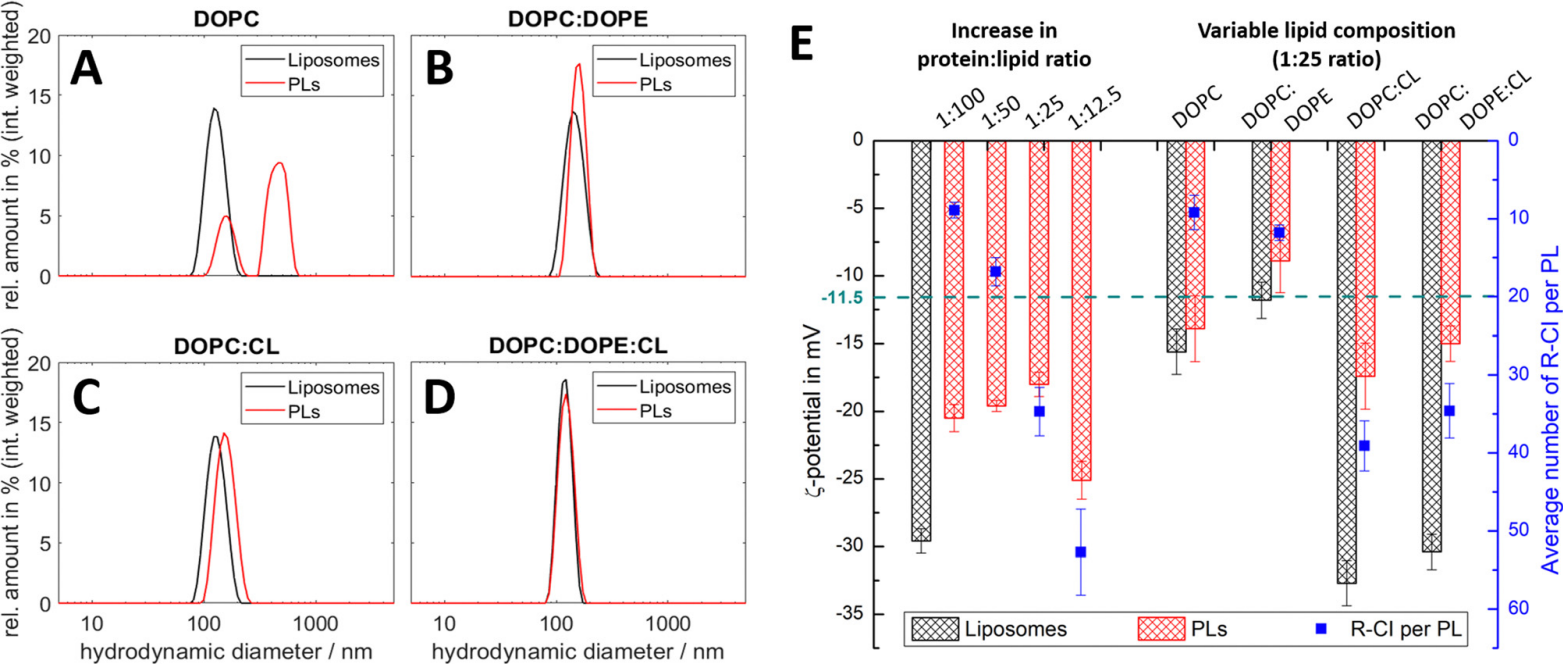
DLS and ELS characterisation of liposomes and PLs with variable lipid composition and R-CI content. (A–D) Intensity-weighted size distributions for liposomes and PLs for lipid systems: DOPC (A), DOPC : DOPE 8.9 : 1.1 (B), DOPC : CL 8.9 : 1.1 (C), DOPC : DOPE : CL 8 : 1 : 1 (D, optimised mixture). The mean diameter relating to the first peak in the polydisperse size distribution of the PLs (A) was used to calculate their *ζ*-potential in E. (E) *ζ*-potentials of liposomes and PLs with increasing protein : lipid ratios from 1 : 100 to 1 : 12.5 (left) and simplified lipid compositions (right), including the average number of reconstituted R-CI per PL (blue data points). The green dashed line is the *ζ*-potential of as-isolated bovine R-CI (10 μg mL^−1^) in aqueous buffer with detergent (no lipids). DOPC : DOPE : CL PLs with protein : lipid ratios of 1 : 25 for the left and right data sets were obtained from different R-CI batches and hence show some variation.

To establish how sensitive the hydrodynamic diameter and *ζ*-potential are to lipid composition and to the amount and orientation of reconstituted protein, we investigated PLs with different lipid compositions and protein : lipid ratios. Fig. 2 summarises the results from DLS and ELS measurements, and the calculated average number of R-CI per PL. The corresponding key biochemical parameters, namely protein retention, orientation and activity, are collated in Table 1.

**Table 1:** Biochemical characterisation of PLs. Measurements of the PLs with increasing protein : lipid ratio were completed on one batch of liposomes (left) and those with variable lipid composition were completed on another batch (right). Comparison between data columns 3 and 8 shows good agreement between PLs prepared with different R-CI batches

Parameter	Change in protein : lipid ratio for DOPC : DOPE : CL PLs				Variable lipid composition (1 : 25 ratio)			
	1 : 100	1 : 50	1 : 25	1 : 12.5	DOPC	DOPC : DOPE	DOPC : CL	DOPC : DOPE : CL
Retention/%	40.6 ± 3.9	42.3 ± 4.0	45.5 ± 3.9	31.7 ± 2.5	12.3 ± 2.1	19.4 ± 0.8	58.4 ± 4.0	53.3 ± 3.0
Orientation/%	82.0 ± 7.8	75.1 ± 6.8	75.0 ± 5.8	70.3 ± 5.3	40.7 ± 6.8	53.1 ± 0.2	71.8 ± 1.8	72.8 ± 3.5
Activity (rel.)^a^/%	94.2 ± 2.2	94.1 ± 2.1	100	81.1 ± 3.1	26.4 ± 7.5	38.9 ± 6.3	96.9 ± 3.1	100

a NADH:O_2_ activities are given as percentage values relative to the optimised composition containing AOX (10 μg mL^−1^) with catalytic rates of 17.9 μmol min^−1^ per mg of R-CI (for DOPC : DOPE : CL PLs, left) and 19.0 μmol min^−1^ per mg of R-CI (for simplified lipid compositions, right). These rates are treated as the maximum coupled rate achieved under optimal reconstitution conditions.

All lipid systems led to liposomes with similar size distribution (data in black, Fig. 2A–D), whereas the influence of the negatively charged CL compared to the zwitterionic DOPC and DOPE^49–51^ is apparent in the *ζ*-potential (black bars, Fig. 2E). As expected, liposomes without CL have a much less negative *ζ*-potential (*ca.* −15 mV) compared to those with CL (*ca.* −30 mV), since CL has two negatively charged phosphate moieties at physiological pH.^52^

DOPC liposomes and DOPC PLs exhibited the same *ζ*-potentials within error (Fig. 2E). The presence of multiple size populations (Fig. 2A) correlates with the poor protein retention (Table 1), indicating ineffective R-CI reconstitution. The second population likely represents merged lipid vesicles. DOPC liposomes and PLs possessed reduced colloidal stability,^53,54^ as is evident from the increased and less uniform size distributions following ELS measurements (Fig. S1, ESI†).

Including DOPE as a non-bilayer forming lipid improves PL formation as shown in the monodisperse size distributions (Fig. 2B). Due to the extended hydrophobic domain (membrane arm) of R-CI, such a non-bilayer forming lipid is probably needed to introduce curvature stress in the vesicles and support the formation of local, transient structures which help to guide R-CI into the bilayer.^55^ The increase in the average number of outward-facing R-CI molecules supports the shift to a more positive *ζ*-potential of the PLs. Nonetheless, the biochemical parameters (Table 1) show that DOPE and DOPC together are not sufficient to support high R-CI catalytic activity.

The previously established ‘optimal’ lipid composition of R-CI PLs with DOPC : DOPE : CL 8 : 1 : 1^14,22^ with a protein : lipid ratio of 1 : 25 (w/w), provides a benchmark for protein activity, outward orientation and retention (Table 1, see also Table S3 (ESI†) for comparison with literature values). At this composition, the *ζ*-potential approximately halves when R-CI is assembled into the liposomes. The *ζ*-potential of the ‘simplified’ DOPC : CL liposomes, which perform equally well in biochemical terms (Table 1), also halved upon reconstitution with R-CI (1 : 25 protein : lipid ratio). The biochemical data show that CL is essential for the reconstitution of catalytically active R-CI,^22^ with its negative charge – as one main contributor to the *ζ*-potential – reporting on the presence of R-CI in liposomes.

In the optimized DOPC : DOPE : CL 8 : 1 : 1 lipid composition, the average number of reconstituted R-CI molecules is proportional to the amount of enzyme added (Fig. 2E). In contrast to PLs with a protein : lipid ratio between 1 : 100 and 1 : 25 that exhibit similar excellent biochemical performance (high R-CI outward orientation, retention and activity), increasing the protein : lipid ratio to 1 : 12.5 negatively impacts protein retention and catalytic activity (Table 1) and breaks the observed trend in *ζ*-potential (see Discussion).

### Influence of CL association to R-CI on catalytic activity

**Fig. 3:**
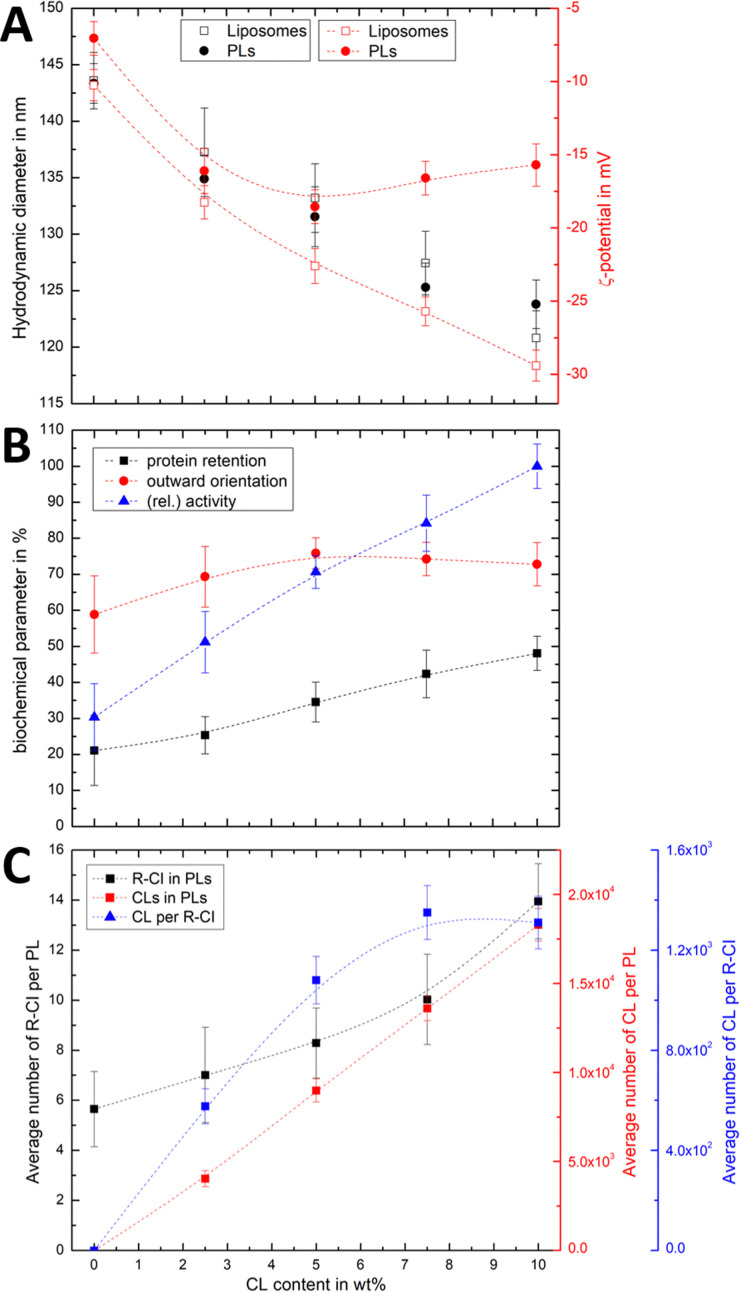
Effect of CL on hydrodynamic diameter, *ζ*-potential and biochemical competence of PLs, and average number of CL per PL and R-CI. (A) Change in hydrodynamic diameter (black) and *ζ*-potential (red) with increasing CL content in PLs (1 : 50 protein : lipid ratio) and liposomes. (B) Biochemical parameters (protein retention, outward orientation, activity) against CL content. The activity, determined for a fixed amount of outward-facing R-CI of 1.5 μg mL^−1^, is given as a relative value compared to the optimised system (DOPC : DOPE : CL 8 : 1 : 1). Data points represent mean values of two batches of PLs prepared with increasing CL content. (C) Effect of increasing CL content on the average number of R-CI in PLs with protein : lipid ratio 1 : 50 (black), with number of CL units in PLs (red) and the average number of CLs per reconstituted R-CI (blue). Dashed lines serve as a guide to the observed trends.

To probe the association of CL with R-CI, we prepared liposomes and PLs with variable CL content while keeping a constant protein : lipid ratio of 1 : 50. Changes in both the hydrodynamic diameter and *ζ*-potential with increasing CL content (Fig. 3A) were compared to the corresponding protein retention, outward-orientation and R-CI activity (Fig. 3B).

For both liposomes and PLs, the mean hydrodynamic diameter decreases linearly with increasing CL content. We attribute this relatively small change (see also Fig. S2, ESI† illustrating the overlap in size distributions) to a decrease in the hydration layer with increasing CL content, rather than a change in the actual (non-experimentally accessible) vesicle size. This interpretation is in agreement with previous studies reporting a decrease in the water permeation of PC membranes with addition of up to 20 mol% CL, which was attributed to a more stable hydrogen-bonding network at the membrane surface.^49^

As expected, increasing the CL content in liposomes resulted in a more negative *ζ*-potential. However, following R-CI reconstitution, after an initial decrease the *ζ*-potential remained approximately constant above 5 wt% CL (Fig. 3A). While protein activity and retention increased linearly with CL content, the trend in outward facing R-CI content mirrors the ‘stagnating’ *ζ*-potential (Fig. 3B), suggesting that above 5 wt% CL, the increasing number of outward-facing R-CI hydrophilic domains dominate the *ζ*-potential.

The vesicle and protein concentrations were further used to understand the average composition of PLs. The amount of CL per vesicle and per protein (Fig. 3C) were determined from the lipid concentration from one PL set with increasing CL content (see Methods and Fig. S3, ESI†). Together with DOPC : DOPE : CL ratios and knowing the average number of R-CI per PL, the average number of CL per PL and per protein could be estimated. In the range of 0–7.5 wt%, the number of CL per R-CI increases linearly and reaches a plateau at 10 wt%, at *ca.* 1300 CL per protein. These high numbers do not represent the actual number of CLs associated with R-CI; only a fraction of the CLs is expected to interact dynamically with R-CI, with the remainder contributing to the entire lipid bilayer system.

The physico-chemical and biochemical characterisation of PLs containing variable amounts of CL show that this negatively charged phospholipid is essential for the effective reconstitution of catalytically active R-CI into the lipid bilayer. Although the outward orientation of R-CI does not improve above 5 wt%, PLs with 10 wt% CL exhibited the overall best biochemical performance. In the Discussion we further analyse the observed trends and information that can be extracted from the *ζ*-potentials in context with the biochemical data.

### *ζ*-Potential measurements detect proton motive force and are sensitive to optimal CL content

Using a quinone recycling system (AOX) for sustained R-CI turnover, we first established the effect of variable AOX concentration on the hydrodynamic diameter and the *ζ*-potential. The outward-facing concentration of R-CI (1.5 μg mL^−1^) and lipid composition (DOPC : DOPE : CL 8 : 1 : 1) were kept constant and the same as in the activity assays. Above 1.0 μg mL^−1^ AOX content, the mean hydrodynamic diameter increases for both liposomes and PLs (Fig. S4, ESI†) and is accompanied by a positive shift in the *ζ*-potential (Fig. S5, ESI†).

**Fig. 4:**
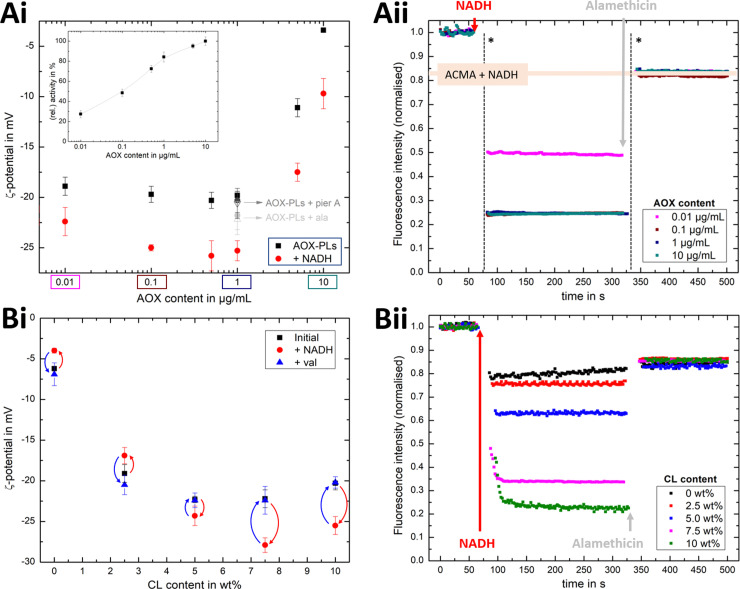
Influence of AOX and CL content on the build-up PMF in PLs. (Ai): changes in the *ζ*-potential with increasing amounts of AOX for PLs with 10 wt% CL (1.5 μg mL^−1^ outward-facing R-CI) before and after injection of NADH (see also Fig. S5, ESI†). Controls with 20 μg mL^−1^ alamethicin (+ala) or 1 μM of piericidin A (+pier A) are shown in grey. NADH:O_2_ activity as a function of AOX content is given in the inset. (Aii) Proton pumping in R-CI PLs with varying AOX content monitored using ACMA fluorescence. All PLs (1.0 μg mL^−1^ outward-facing R-CI) were treated with 0.1 μM valinomycin. Proton pumping was initiated with 500 μM of NADH and addition of 10 μg mL^−1^ of alamethicin led to the collapse of the PMF. (Bi) Comparison of *ζ*-potential for PLs (1.5 μg mL^−1^ outward-facing R-CI) with varying CL content under the influence of AOX (1 μg mL^−1^). NADH induced the formation of a PMF, subsequently collapsed with the addition of valinomycin. (Bii) Proton pumping in R-CI PLs (+1 μg mL^−1^ AOX) with increasing CL-content monitored using ACMA fluorescence. Other conditions were as in (Aii).

Fig. 4Ai shows that the established PMF, initiated by NADH addition, is visible at all AOX concentrations through the negative shift in *ζ*-potential, reflecting the transfer of positive charge (H^+^) from the outside to the inside of the PLs. At very low AOX content (0.01 μg mL^−1^), the small change in *ζ*-potential suggests that insufficient AOX is associated to the R-CI PLs to re-oxidise the quinone pool effectively and hence support build-up of a steady PMF across the lipid bilayer.

ACMA fluorescence quenching assays were performed to assess ΔpH at variable AOX concentration (Fig. 4Aii), and mirror the ELS results in Fig. 4Ai. Control experiments with porous AOX-containing PLs created by the addition of alamethicin, or inhibition of the quinone-binding site in R-CI with piericidin A, led to no significant change in the *ζ*-potential after the addition of NADH (Fig. 4Ai). The slight decrease in *ζ*-potential after the addition in alamethicin can be explained by an enhanced lipid scrambling in the bilayer due to the inclusion of peptides, affecting the amount of CL in the outer liposome leaflet.^56^

The change in *ζ*-potential as a result of the established PMF (Fig. 4Ai) does not correlate with the trend in R-CI catalytic activity as a function of AOX content (Fig. 4Ai inset). Instead, it correlates with the formation of Δ*ψ* (see Discussion). Δ*ψ* builds up very quickly, but is then opposed by proton leakage, which increases in response. In steady-state, both the proton pumping and leakage are equal and, provided proton pumping is substantial, the Δ*ψ* at which the steady-state is established is not strongly dependent on the rate of pumping (*i.e.* activity).

Having established that *ζ*-potentials are sensitive to Δ*ψ* of the PMF even at low levels of enzyme activity, we proceeded to investigate the influence of CL. PLs with a fixed AOX concentration of 1 μg mL^−1^ were chosen to minimize the impact of aggregated AOX species (Fig. S4, ESI†) on ELS measurements. Given that addition of NADH led to build-up of a PMF and hence a negative shift in the *ζ*-potential at 10 wt% CL (Fig. 4Bi), the subsequent addition of valinomycin (to allow compensatory movement of K^+^ ions, see Fig. S6, ESI†) should dissipate the Δ*ψ* and return the effective surface charge to its original value. This expected trend was indeed observed for AOX-PLs with a CL content of or above 5 wt% (Fig. 4Bi). Surprisingly, lower CL contents led to the opposite behaviour, with a decrease in *ζ*-potential upon NADH addition, albeit restoration of the initial (higher) value upon dissipation of Δ*ψ* was still observed (Fig. 4Bi, see also Fig. S7, ESI†). In contrast, ΔpH as monitored by ACMA quenching in the presence of valinomycin (Fig. 4Bii), increased with increasing CL content broadly in line with the corresponding increase in R-CI activity (Table S7, ESI†), but was also likely affected by the integrity of the membrane. At low CL content (below 5 wt%), PLs appear to be leaky. Control measurements with 2.5 wt% AOX-PLs in the presence of the R-CI inhibitor piericidin A did not exhibit a significant shift in *ζ*-potential after addition of NADH and valinomycin (see Fig. S7, ESI†). The utility of using *ζ*-potentials to assess the PMF is discussed further below.

## Discussion

### Correlation between changes in *ζ*-potential and biochemical parameters

**Fig. 5:**
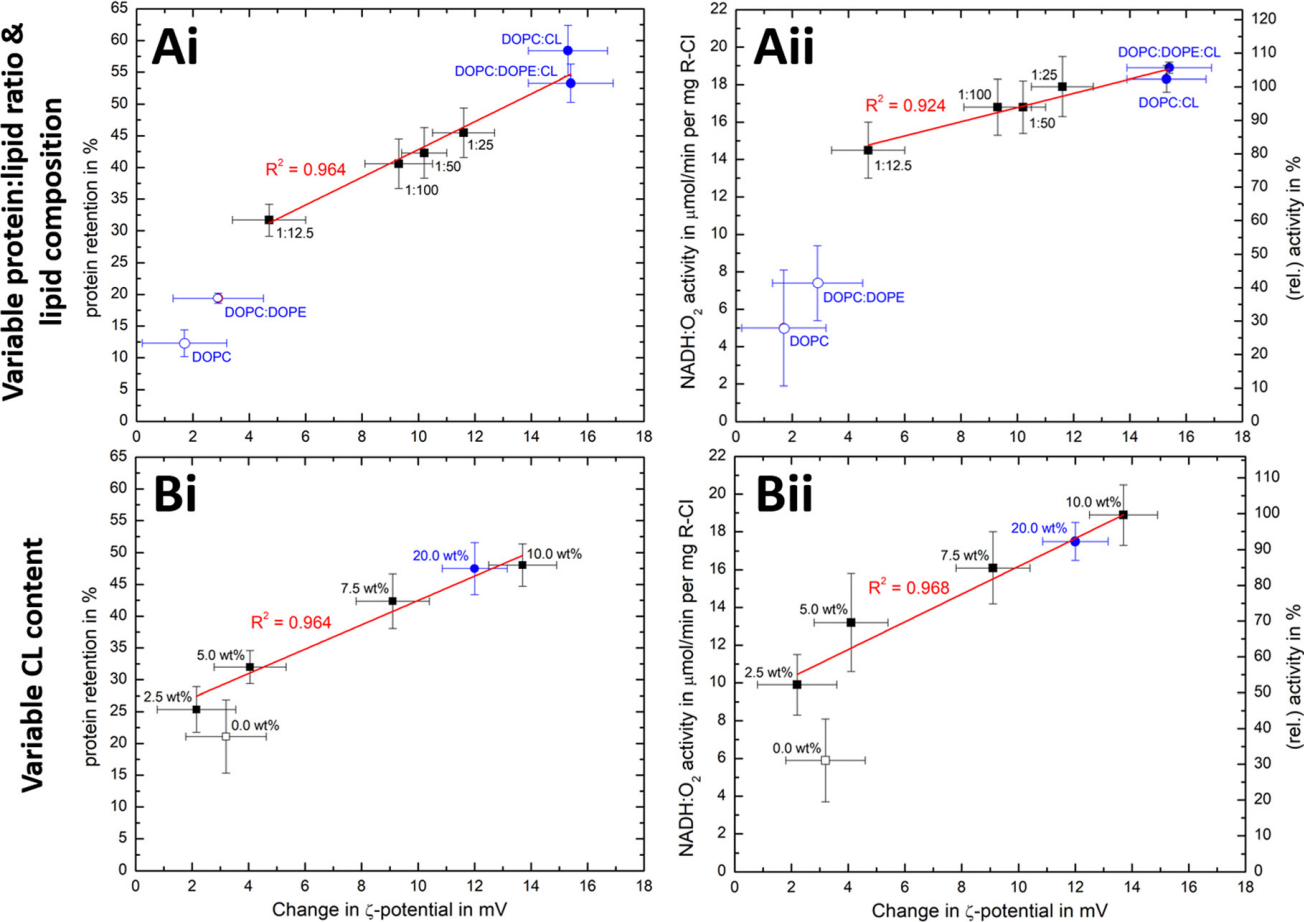
Correlation of *ζ*-potential changes with biochemical parameters. Plots of Δ*ζ* from PLs with (A) increase in protein : lipid ratio (fixed 10 wt% Cl content) and variable lipid composition and (B) increased in CL content (fixed 1 : 50 protein : lipid ratio) against protein retention (i) and catalytic activity (ii). The relative activity values use (A) protein : lipid ratio 1 : 25 or (B) the 10 wt% CL data as reference point. Data points for Ai/Aii are taken from Fig. 2E and Table 1, whereas Bi/Bii are the mean values for two measured PLs sets (see Fig. S8, ESI† for individual data sets). Data points linked to lipid mixtures without CL are present as hollow spheres or circles. The primary data for the 20 wt% CL sample can be found in Table S9 (ESI†).

We have seen that the *ζ*-potential difference between liposomes and PLs depends on lipid composition and on the ability to reconstitute R-CI (Fig. 2E and 3B). To better understand to what extent *ζ*-potentials correlate with biochemical parameters and to allow a meaningful analysis independently from the lipid mixture, we looked at changes in *ζ*-potential, **Δ*****ζ*** = ***ζ*****(PLs) −*****ζ*****(liposomes)**, as shown in Fig. 5.

Δ*ζ* exhibits an approximately linear relationship with protein retention (Fig. 5Ai and Bi) and activity (Fig. 5Bi and Bii). Data points linked to lipid systems missing CL (hollow symbols in Fig. 5) do not follow the linear trends, reflecting the essential role of CL for effective reconstitution, as well as the need for its negative charge to lead to significant Δ*ζ* values.

There is also a linear trend between Δ*ζ* and the number of outward-facing R-CI molecules, for protein : lipid ratios up to 1 : 25 and fixed 10 wt% CL (Fig. S9A, ESI†) as well as variable CL content (Fig. S9B, ESI†). An increased number of hydrophilic domains facing into the bulk solution thus induces a larger positive Δ*ζ*. Comparing Δ*ζ* with the number of outward-facing R-CI for DOPC : DOPE lipid mixtures and the ‘optimised’ composition at a protein : lipid ratio of 1 : 25 shows that ∼70% of Δ*ζ* can be attributed to the hydrophilic domains of R-CI facing into the bulk solution (see ESI† Section 4.2). The remainder is attributed to protein–lipid interactions in the hydrophobic domain, mainly between CL and the positively charged areas (see electrostatic surface charge of bovine R-CI in Fig. S10, ESI†).

At the high protein : lipid ratio of 1 : 12.5 (Fig. 5Ai, ii), Δ*ζ* does not increase further and the biochemical performance drops. Protein-protein interactions between R-CI molecules probably start to dominate, affecting protein–lipid interactions especially with CL. A similar effect was reported for light-harvesting complexes, where increased protein concentrations induced fluorescence quenched states in PLs.^57^ Moreover, increasing the CL content to 20 wt% did not improve retention and activity further (Fig. 5Bi, ii, see also Table S9, ESI†). Even though we cannot be sure of the reason for the worsened biochemical performance, Δ*ζ* correlates with it.

Overall, the magnitude of Δ*ζ* is an indicator of R-CI biochemical performance, especially when CL is present as a sensitive ‘reporter’ lipid. Calibration with biochemical assays is required to extrapolate any quantitative information on biochemical performance from Δ*ζ* and the direct proportionality only holds when CL is present. Based on light scattering experiments investigating membrane asymmetry,^58^ we anticipate that other negatively charged phospholipids such as phosphatidylglycerol would also give rise to a sufficiently large Δ*ζ* to enable at least qualitative predictions on the biochemical competence of the vesicles.

### Average number of reconstituted R-CI and association to CL

We provided values for the average number of reconstituted R-CI per PL by combining information on the number of vesicles and protein retention. For a 1 : 25 protein : lipid ratio and the optimised lipid composition (DOPC : DOPE : CL 8 : 1 : 1), an average of 35 R-CI molecules per PL was determined. This value is comparable with previous calculated estimates on a similar system.^22^ However, individual PLs within a given sample (produced by extrusion methods) differ in size/curvature, permeability, lipid composition, protein content and orientation of reconstituted proteins.^59–61^ Values such as the number of CLs or R-CI per PL should be considered indicative rather than absolute given the heterogeneity of the sample.

For the ‘optimised’ lipid composition at protein : lipid ratios of 1 : 25, where Δ*ζ* is dominated by the number of outward-facing hydrophilic R-CI domains, we estimated that an average of 67 CLs are ‘associated’ with one R-CI (Table S7, ESI†). Previous studies identified that ∼10 CL lipids co-purify with isolated bovine R-CI,^62^ and 9 well-ordered CL were modelled in the mouse R-CI structure.^63^ Interestingly, in coarse-grained molecular dynamics (MD) simulations that considered the membrane environment of *Thermus thermophilus* R-CI, the number of bound CL was found to be significantly higher (40–50 : 1),^64^ more closely aligned with our estimated ‘associated’ CL values. The membrane arm of bovine R-CI in lipid nanodiscs was found to contain an average of 295 different phospholipids.^65^ The ‘associated’ CLs we determined may thus be better described as being part of a functional paralipidome,^66^ a wider local environment that is however distinct from the bulk.

The question remains as to how CL fulfils its essential role. Molecular dynamics simulations with vesicles containing POPC, DOPE and CL found that even just 1 wt% CL led to reduced lateral diffusion of lipid molecules and reduced membrane fluidity.^67^ Moreover, CL is known to act as a proton trap in membranes that conduct oxidative phosphorylation.^68^ Cardiolipin is therefore crucial for the transport of ions and small molecules through the lipid bilayer.

### Visualising PMF *via ζ*-potential measurements

**Fig. 6:**
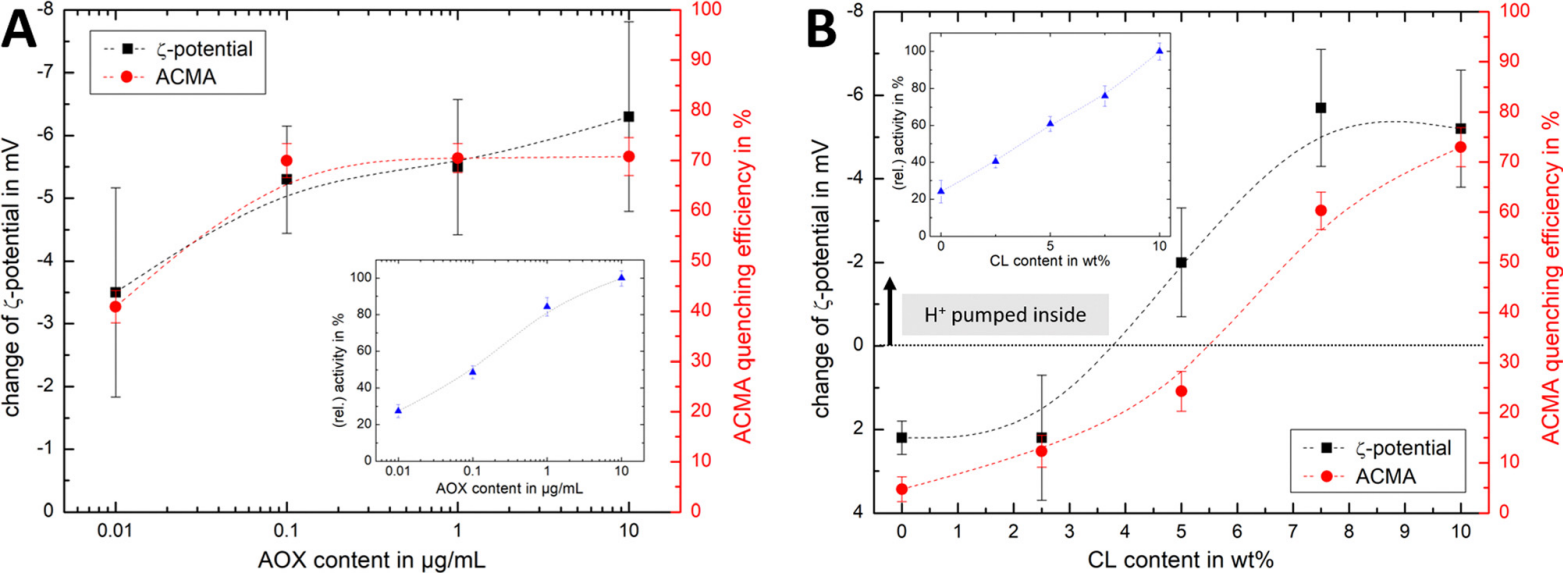
Comparison of ELS and ACMA measurements. Comparison of the change in *ζ*-potential for AOX-R-CI PLs before and after initiating proton pumping *via* NADH addition with the ACMA quenching efficiency, with (A) showing variable AOX content with fixed 10 wt% CL (linked to Fig. 4Ai and Aii) and (B) showing variable CL content (linked to Fig. 4B). Dashed lines in all plots serve as guide for the observed trends. Insets show the change in relative activity as determined with the NADH:O_2_ assay. The *y*-axis for the *ζ*-potential change was flipped (from positive to negative values) to allow easier comparison.

Changes in *ζ*-potential, **Δ*****ζ***′ = ***ζ*****(AOX-PLs + NADH)** − ***ζ*****(AOX-PLs)**, and the established ACMA fluorescence quenching assays of AOX-PLs pumping protons are compared in Fig. 6. ELS measurements offer the advantage that no ‘additives’ are required. In contrast, established ACMA and oxonol VI^69^ assays require the addition of a fluorophore. In ACMA measurements, the choice of the right concentration is crucial to avoid *e.g.* initial self-quenching due to dimerization.^70^ Nonetheless some precautions need to be taken for successful ELS measurements: (1) large numbers of vesicles, leading to vesicle-vesicle interactions or multiple scattering effects,^34^ must be avoided; (2) the strength of the applied alternating electric field needs to be adjusted carefully to prevent Joule heating;^38^ (3) for newly studied vesicle systems, size distributions should be compared before and after ELS experiments (see *e.g.* Fig. S1 and S6, ESI†) to guarantee the integrity of the samples tested.

Similar trends for both ACMA and Δ*ζ*′ measurements (Fig. 6A) are observed upon increasing the AOX content with fixed CL content. The Δ*ζ*′ data point at 10 μg mL^−1^ AOX is an outlier, due to the increased hydrodynamic diameter of the vesicles (as shown in Fig. S4B, ESI†) affecting the scattered light. For lipid mixtures with increasing CL content (Fig. 6B), we thus fixed the AOX content for both *ζ*-potential and ACMA measurements to 1 μg mL^−1^. Although the overall trend is similar for both techniques, and Δ*ζ*′ measurements are a suitable method to access the build-up (and collapse) of the PMF in AOX-PLs, there are some interesting discrepancies. First, it is important to emphasise that ACMA is a measure of ΔpH alone, whereas Δ*ζ*′ captures changes in potential at the slipping plane (Fig. 1), which reflect Δ*ψ* but are also sensitive to pH^71^ and ionic strength. Second, the reduced lateral diffusion and membrane fluidity introduced by CL^67^ (which is amplified at low wt% CL) might negatively impact the transport of ACMA across the membrane, leading to a reduced measurement efficiency compared to Δ*ζ*′ (Fig. 6B). Like Δ*ζ* for biochemical performance, the magnitude and direction of Δ*ζ*′ thus serves as a useful guide for the PMF built up by R-CI.

## Conclusions

We characterised R-CI reconstituted into liposomes using both light scattering methods and biochemical assays. We have shown that whilst there is no clear trend with absolute *ζ*-potentials, there is a linear correlation in the changes in *ζ*-potential Δ*ζ* (PLs *vs.* liposomes) with key biochemical characteristics, namely NADH:O_2_ oxidoreduction activity and protein retention. Δ*ζ* as an indicative tool for the biochemical competence of PLs was thus contingent on the presence of the strongly charged lipid CL. Generally, we established that the larger Δ*ζ*, the better the biochemical performance. Although our focus was on the functionally important CL in this work with R-CI, we speculate that other charged lipids such as phosphatidylglycerol (PG), phosphatidylserine (PS) or phosphatidylinositol (PI) would give rise to a similar behaviour and that Δ*ζ* as a physical guide to biochemical performance may be useful for other PL systems that do not solely contain zwitterionic lipids. Δ*ζ*′ further emerged as a useful tool to assess the PMF in PLs. Although calibration measurements with biochemical essays are clearly necessary to interpret Δ*ζ* or Δ*ζ*′ beyond qualitative terms, the magnitude (and direction) of Δ*ζ* (or Δ*ζ*′) served as a guide to the performance of PLs, and may prove to be useful for proteins for which biochemical assays are not available.

ELS measurements further indicated that CL is essential for membrane integrity and in preventing leakage of small ions. We envisage that ELS may be used quantitatively to determine the number of ‘associated’ CL per R-CI and the impact of the hydrophilic domain of R-CI on changes of the *ζ*-potential as it becomes possible (*e.g. via* microfluidics^72^) to generate more homogeneous PLs than is possible with extrusion methods. In recent studies, ELS measurements enabled quantification of the asymmetric uptake of negatively charged lipids in a label-free and non-destructive assay.^58^*ζ*-potentials could prove to be invaluable in informing on the lipid composition of the outer leaflet of artificial membrane systems,^73^ helping to explore the effects of membrane asymmetry on R-CI.

## Author contributions

JE performed all research and data analysis, except for purification of bovine complex I and AOX, which was performed by JJW. MMR and JE designed the research with contributions from JH and JJW. As hosts of JE's fellowship, MMR and JWE directed the research. JE and MMR wrote the manuscript with input from JH, JJW and JWE. All authors read and approved the final manuscript.

## Conflicts of interest

There are no conflicts to declare.

## Supplementary Material

CB-004-D2CB00158F-s001
