# Supplementary material for: Using light scattering to assess how phospholipid–protein interactions affect complex I functionality in liposomes
Source: RSC Chem Biol. 2023 Mar 20;4(6):386–98. doi: 10.1039/d2cb00158f (PMC10246558; doi:10.1039/d2cb00158f)
Supplement: CB-004-D2CB00158F-s001 [file CB-004-D2CB00158F-s001.pdf]

Electronic Supplementary Material (ESI) for RSC Chemical Biology.
This journal is © The Royal Society of Chemistry 2023

Supporting Information

Using light scattering to assess how phospholipid-protein interactions affect complex I functionality in liposomes

Jana Eisermann[a], John J. Wright[b], James Wilton-Ely[a]*, Judy Hirst[b], Maxie M. Roessler[a]*

[a] Department of Chemistry, Imperial College London, Molecular Sciences Research Hub, White City Campus, London W12 0BZ, UK

[b] The Medical Research Council Mitochondrial Biology Unit, University of Cambridge, The Keith Peters Building, Cambridge Biomedical Campus, Cambridge CB2 0XY, UK

Contents

1 Overview of Parameters 2
2 Characterisation of liposomes and PLs 3
2.1 Lipid compositions in liposomes and PLs 3
2.2 Comparison of physical and biochemical data on different batches of PLs and with literature values 4
3 Principles of DLS and ELS methods 5
3.1 Introduction to Multi-Angle Dynamic Light Scattering (MADLS) 5
3.2 Calculating the number of vesicles using MADLS 6
3.3 Determining the lipid concentration in liposomes and PLs via light scattering 6
3.4 Measurement and calculation of the ζ–potential 8
4 Derivations for calculated values 10
4.1 Average number of cardiolipins and R-CI per proteoliposome 10
4.2 Contributions to Δζ and average number of 'associated' CLs to reconstituted 11
4.3 Influence of AOX and CL content on R-CI activity 12
5 Supplementary Figures 12
5.1 Intensity-weighted size distributions for liposomes and PLs before and after performing ELS measurements 12
5.2 Vesicle size distributions with increasing CL content 13
5.3 Lipid concentration results 13
5.4 Aggregation of AOX and influence on liposomes 13
5.5 Check integrity of PLs after proton pumping measurements with ELS 15
5.6 Change in ζ-potential with addition of NADH and valinomycin 16
5.7 Correlation between ζ-potential and biochemical parameters 17
5.8 Electrostatic charge on the surface of R-CI 19
6 References 20

1 Overview of Parameters

Table S1 – Overview over the parameters used in the main manuscript. For each parameter, the symbol and unit are given together with the method/technique applied to derive them.

Parameter	Symbol	Unit	Method	Notes
Hydrodynamic diameter	d_H	nm	MADLS	
Vesicle diameter/radius	d/a	nm	-	Vesicle radius included in Henry equation to calculate ζ-potential
Zeta potential	ζ	mV	ELS	Focus on changes in zeta potential:<br>$\Delta\zeta = \zeta(\text{PLs}) — \zeta(\text{liposomes})$<br>$\Delta\zeta' = \zeta(\text{AOX-PLs+NADH}) — \zeta(\text{AOX-PLs})$
Total lipid concentration	c_{ilpid}	mg/mL	DLS, Stewart assay	
Protein retention	-	%	NADH:APAD$^+$	
Protein concentration	$c_{R\text{-}CI}$	µg/mL	NADH:APAD$^+$	Calculated based on protein retention value
Outward orientation	-	%	NADH:APAD$^+$	Compare results with and without alamethicin
Coupled activity	-	µmol/min per mg R-CI	NADH:O_2	rel. activity values (in %) use the value for optimised lipid mixture as reference (100% activity)
Number of vesicles	N_{ves}	-	MADLS	Raw data measured in number of vesicles per mL (data given in the manuscript adjusted based on used sample volume)
Number of CL per PL	-	-	-	Based on total lipid concentration, the number of vesicles and the lipid composition (%wt of CL in mixture)
Average number of R-CI per PL	-	-	-	Calculated based on protein and number of vesicles

2 Characterisation of liposomes and PLs

2.1 Lipid compositions in liposomes and PLs

For all experiments using a simplified lipid mixture or adjusting the cardiolipin content, the final amounts for each component are summarised in **Table S2**.

Table S2 - Overview of the prepared lipid mixtures for liposomes and PLs. The compositions are characterised by their respective mass and molar ratio.

		DOPC	DOPE	CL
'Optimal'	Mass ratio	8.00	1.00	1.00
(10 wt% CL)	Molar ratio	7.57 / 15.28	1.00 / 2.02	0.50 / 1.00
DOPC	Mass ratio	1.00	-	-
	Molar ratio	1.00	-	-
DOPC + DOPE	Mass ratio	8.89	1.11	-
(0 wt% CL)	Molar ratio	7.58	1.00	-
DOPC + CL	Mass ratio	8.89	-	1.11
	Molar ratio	15.30	-	1.00
2.5 wt% CL	Mass ratio	8.67	1.08	0.25
	Molar ratio	7.58	1.00	0.11
5.0 wt% CL	Mass ratio	8.44	1.06	0.50
	Molar ratio	7.58	1.00	0.23
7.5 wt% CL	Mass ratio	8.22	1.03	0.75
	Molar ratio	7.57	1.00	0.36
20 wt% CL	Mass ratio	7.11	0.89	2.00
	Molar ratio	7.56	1.00	1.11

2.2 Comparison of physical and biochemical data on different batches of PLs and with literature values

Before analysing the influence of different lipid environments, we compared the biochemical parameters of our PLs with DOPC:DOPE:CL 8:1:1 (**Table S3**; averaged over four different aliquots of purified bovine complex I) with results from previous studies.

Table S3 - Comparison of all determined physical and biochemical parameters for four PL batches (protein:lipid ratio 1:25) prepared with different aliquots of purified bovine complex I. Mean values with standard deviations from the main text are also included. For comparison, values of 48.0±3.0 %, 74.0±4.0 % and 21.4±2.0 µmol/min per mg R-CI have been reported previously for protein retention, orientation and activity, respectively.[1]

Parameter	Batch Nr. 1	Batch Nr. 2	Batch Nr. 3	Batch Nr. 4	Mean
d_H (liposome) in nm	120.5 ± 2.2	130.3 ± 2.2	119.5 ± 1.9	119.9 ± 1.5	**122.6 ± 5.2**
ζ(liposome) in mV	-30.5 ± 1.8	-30.4 ± 1.3	-29.5 ± 1.1	-28.8 ± 0.3	**-29.8 ± 0.8**
N_{ves}(liposome)	3.66 ± 0.40 x 10^{11}	2.24 ± 0.17 x 10^{11}	1.56 ± 0.11 x 10^{11}	4.18 ± 0.22 x 10^{11}	**2.91 ± 1.05 x 10^{11}**
d_H (PLs) in nm	134.7 ± 1.5	139.3 ± 2.3	136.8 ± 2.0	123.6 ± 2.2	**133.6 ± 6.9**
ζ(PLs) in mV	-15.4 ± 1.8	-15.0 ± 1.3	-15.0 ± 1.6	-14.0 ± 1.1	**-14.9 ± 0.6**
N_{ves} (PLs)	3.09 ± 0.62 x10^{10}	3.62 ± 0.53 x 10^{10}	3.51 ± 0.12 x 10^{10}	4.42 ± 0.26 x 10^{10}	**3.66 ± 0.48 x 10^{10}**
retention in %	46.1 ± 4.5	53.0 ± 3.0	58.9 ± 3.5	51.3 ± 3.7	**52.3 ± 5.3**
orientation in %	72.6 ± 5.0	72.8 ± 3.8	83.1 ± 3.0	83.0 ± 3.2	**77.9 ± 6.0**
activity in µmol/min per mg	18.0 ± 2.5	17.9 ± 2.2	22.5 ± 1.3	21.2 ± 1.8	**19.6 ± 2.0**

d_H = hydrodynamic diameter; ζ = zeta potential; N_{ves} = number of vesicles

3 Principles of DLS and ELS methods

3.1 Introduction to Multi-Angle Dynamic Light Scattering (MADLS)

Multi-angle dynamic light scattering (MADLS) provides an angular-independent vesicle size distribution with improved resolution relative to standard DLS techniques measuring at a single angle.[2] As populations may appear weakly scattering at one detection angle, but more apparent at a different one, the combination of all measured size distributions improves insight into all vesicle sizes present in the sample.

The Zetasizer Ultra performs an automated series of single-angle measurements: backscatter (173°), side scatter (90°) and forward scatter (15°). MADLS derives a single number of vesicles for all detection angles by analysing multiple autocorrelations which are treated as parallel observations. The goal is to find the size distribution that is the best fit for all data sets. The additional time investment to perform MADLS (~5 min for three replicates) instead of single-angle light scattering (~2 min for three replicates) is minimal.

Single-angle DLS measurements are analysed by applying a least square minimization algorithm on the recorded autocorrelation function to determine the best fit and the related vesicle distribution. The specific algorithm (here: "General purpose") influences the constraints that may be placed on the solution (e.g. smoothness or non-negativity). The transformation from result to measurement can be described by the following scattering matrix

$$\boldsymbol{g_1}(\tau) = \boldsymbol{K}(\tau, d)\boldsymbol{x}(\mathrm{d}). \qquad \text{Eq. 1}$$

The autocorrelation coefficient $\boldsymbol{g_1}(\tau)$ is measured at each lag time τ, whereas the number of vesicles $\boldsymbol{x}(\mathrm{d})$ is intrinsically weighted by the intensity of the scattered light of each component. Each element in the scattering matrix, $\boldsymbol{K}(\tau, d)$ can be calculated as a function of lag time and vesicle size distribution according to

$$g_1(\tau) = \exp(-q^2 D_\mathrm{t} \tau), \qquad \text{Eq. 2}$$

where the field autocorrelation function $g_1(\tau)$ is further defined by the scattering vector q and the translational diffusion coefficient D_t. The latter corresponds to the vesicle size (hydrodynamic diameter d_H) via the Stokes equation.

MADLS extends the single-angle method by transforming equation 1 into a system containing multiple linear equations

$$\begin{bmatrix} \boldsymbol{g_1}(\theta_1, \tau) \\ \boldsymbol{g_2}(\theta_2, \tau) \\ \boldsymbol{g_3}(\theta_3, \tau) \end{bmatrix} = \begin{bmatrix} \boldsymbol{K}(\theta_1, \tau, d) \\ \boldsymbol{K}(\theta_2, \tau, d) \\ \boldsymbol{K}(\theta_3, \tau, d) \end{bmatrix} \boldsymbol{x}(\mathrm{d}), \qquad \text{Eq. 3}$$

where $\theta_1, \theta_2, \text{and } \theta_3$ denote the three applied scattering angles. One crucial aspect when combining data from several angles is to preserve the weighting inherent in the vesicle size distribution. Therefore, the scattering matrix for each measurement angle is weighted by the expected scattered intensity. The scattered intensity (specifically differential scattering cross-section) is calculated using Mie theory[3] and requires knowledge of the material and dispersant optical properties (refractive index and absorption).

In deriving the solution, the least squares minimisation algorithm aims to reduce the magnitude of the residual (the difference between the measured autocorrelation function and the prediction).

Each angular contribution to the residual is weighted separately to account for the result uncertainty expected for that detection angle.

3.2 Calculating the number of vesicles using MADLS

The size distribution produced by MADLS applies an array of discrete size classes logarithmically spaced across the measurable size range to represent the distribution of sizes within the sample. Each vesicle contributes to the result by an amount proportional to the intensity of light that it scatters; the respective scattered intensity is, therefore, a function of the vesicle diameter *d.* As such, the native DLS-derived vesicle size distribution is termed an intensity-weighted size distribution *x*(d). The intensity-weighted size distribution is related to the absolute number of vesicle distribution, *ρ*(d), i.e. the number of vesicles per unit volume per size class, according to equation 4[4]:

$$p(\mathrm{d}).\frac{\mathrm{d}C_{\mathrm{sca}}}{\mathrm{d}\Omega}(\mathrm{d}) = x(\mathrm{d}) \cdot I_{\mathrm{par}} \frac{R_{\mathrm{tol}}}{I_{\mathrm{tol}}}, \qquad \text{Eq. 4}$$

where $\frac{\mathrm{d}C_{\mathrm{sca}}}{\mathrm{d}\Omega}(\mathrm{d})$ describes the size-dependent differential scattering cross-section of the vesicles, i.e. the fraction of photons scattered into a unit solid angle, I_{par} is the derived photon count rate due to the vesicle scattering, I_{tol} is the derived photon count rate scattered by the reference liquid toluene. R_{tol} is the Rayleigh ratio (differential cross-section per unit volume) of toluene. The differential scattering cross-section is calculated applying Mie theory[3], therefore knowledge about the material and dispersant refractive index are needed.

The left-hand side of the equation can be interpreted as total light scattered by each vesicle size class. The right-hand side, the detected photon count rate, is normalised by the instrument detection efficiency defined via the detected count rate per unit volume for a toluene reference sample. The required information and measurements to derive the number of vesicles via the ZS Xplorer software are listed below:

- Backscatter-equivalent intensity-weighted size distribution using MADLS for a given sample
- Calculated derived mean count rate for each sample corrected by subtracting the contribution from the dispersant
- Derived mean count rate dispersant (10 mM MOPS, pH 7.4, 50 mM KCl): 36.8 kcps
- Toluene derived mean count: $I_{\mathrm{tol}} = 907\ \mathrm{kcps}$
- Toluene Rayleigh ratio: $R_{\mathrm{tol}} = 1.34 \text{ x } 10^{-5}\ \mathrm{cm}^{-1}$
- Material characteristics (liposomes): refractive index 1.45, absorption 0.001

3.3 Determining the lipid concentration in liposomes and PLs via light scattering

Based on the approach introduced by Di Prima et.al.[5], the essential parameters that need to be fixed for the two performed extrusions are:

- Weighted total mass of lipid components employed for preparation of the starting film (M_0)
- Recovered volumes after the first and second extrusion cycle (V_1, V_2)
- Total volume inserted into extruder (V_T), i.e. the sum of the starting volume and the volume of solvent which is added for second extrusion. Note that V_T will not correspond to the sum $V_1 + V_2$, since the dead volume ΔV remains trapped.

By measuring the volume obtained after the second extrusion, we could determine the dead volume of the setup.

To calculate the actual weight concentration in our samples, the light scattering intensity on appropriate dilutions of the two extruded solutions (I and II) is measured. The static light scattering intensity measured at a specific detection angle θ

$$I = kM_{\mathrm{w}}cP_{\theta} \qquad \text{Eq. 5}$$

is linked to the weight averaged molecular mass (M_{w}), the weight concentration (c) and the so-called form factor (P_{θ}). The constant k depends on the experimental setup and sample parameters. Equation 5 is valid for diluted conditions, where particle interactions are absent. Moreover, the form factor can be approximated to 1 if the dimension of the studied particles is smaller than 1/10 of the laser wavelength used.

For the extruded samples I and II, the light scattering is measured with diluted samples (dilution factors d_1 and d_2) and can be expressed as:

$$I_1 d_1 = kM_{\mathrm{w}}c_1P_{\theta}, \qquad \text{Eq. 6.1}$$

$$I_2 d_2 = kM_{\mathrm{w}}c_2P_{\theta}. \qquad \text{Eq. 6.2}$$

For the two samples, the parameters k, M_{w} and P_{θ} are the same. Since the form factor depends on the vesicle size distribution, MADLS measurements are used to compare the distribution between I and II to ensure this approach is applicable for the prepared samples.

The two equations 6.1 and 6.2 can be combined into:

$$\frac{I_1 d_1}{I_2 d_2} = \frac{c_1}{c_2}, \qquad \text{Eq. 7}$$

which contains two unknown variables with c_1 and c_2. However, based on the extrusion experiment, we can describe how the lipid mass is conserved by

$$M_0 = c_1V_1 + c_2(V_{\mathrm{T}} - V_1), \qquad \text{Eq. 8}$$

applying the correlation of $V_2 + \Delta V = V_{\mathrm{T}} - V_1$. In the calculations, it is assumed that the concentration in volume V_2 and ΔV corresponds to c_2 (since the amount of spare lipids trapped in the extruder is negligible). Equations 7 and 8 can be combined to give:

$$\frac{M_0}{V_1} - \frac{c_2(V_T - V_1)}{V_1} = c_2\frac{I_1 d_1}{I_2 d_2}. \qquad \text{Eq. 9}$$

With the following simplifications: $\alpha = \frac{M_0}{V_1}$, $\upsilon = \frac{(V_{\mathrm{T}} - V_1)}{V_1}$ and $\gamma = \frac{I_1 d_1}{I_2 d_2}$, the concentrations of interest can be calculated using:

$$C_2 = \frac{\alpha}{\gamma + \nu}, \qquad \text{Eq. 10}$$
$$C_1 = \alpha - \nu C_2 = \frac{\alpha\gamma}{\gamma + \nu}.$$

The method presented is applicable to vesicles of various dimensions and the signal can be collected at any detection angle for the two samples. In case of the Zetasizer Ultra, data measured at back and side scattering were used for the lipid concentration calculations.

For our PLs samples, the same calculation was applied, but instead of comparing the two extruded samples, extrusion I was correlated with the PL data.

3.4 Measurement and calculation of the ζ–potential

Principle

Calculation of the ζ-potential involves the measurement of electrophoretic mobility μ_e as well as knowledge of solvent viscosity, ionic strength, and vesicle size. Under an applied electrical field, charged vesicles move due to their ζ-potential, which cannot be measured directly and is deduced from μ_e.[6] The electrophoretic mobility (equation 11) itself is defined as:

$$\mu_\mathrm{e} = \frac{v}{E}, \qquad \text{Eq. 11}$$

with v as the velocity of the charged vesicles and E as electric field strength. By electrophoretic light scattering (here Phase Analysis Light Scattering; PALS), the speed of the mobile vesicles is determined through the frequency shift between the light scattered by the sample and the original laser (Doppler shift).[7] The ζ-potential is calculated with the measured electrophoretic mobility μ_e using Henry's equation:

$$\mu_\mathrm{e} = \frac{2\varepsilon_0\varepsilon_\mathrm{r}\zeta F(\kappa a)}{3\eta}, \qquad \text{Eq. 12}$$

where ε_0 = vacuum permittivity, ε_r = relative permittivity, $F(\kappa a)$ = Henry's function and η = viscosity. Henry's function itself depends on the thickness of the electrical double layer κ^{-1} and the vesicle radius a, which we here replace with the mean value of the measured hydrodynamic radii R_H for each vesicle system. To determine the actual ζ-potential values, we used the relative permittivity and viscosity of pure water directly from the ZS Xplorer database included with the analysis software. The thickness of the electrical double layer κ^{-1}, also known as the Debye length, can be calculated as follows:

$$\kappa^{-1} = \sqrt{\frac{\varepsilon_0\varepsilon_\mathrm{r}k_\mathrm{B}T}{2e^2 I N_\mathrm{A}}}, \qquad \text{Eq. 13}$$

where k_B = Boltzmann constant, T = temperature, e = the elementary charge, N_A = the Avogadro's number and I = the ionic strength. The ionic strength of each sample depends on the concentration c_i and charge z_i of the ionic building units and can be determined using $I = \frac{1}{2}\sum c_\mathrm{i} z_\mathrm{i}^2$. The ionic strength of the aqueous buffer solution is defined by the concentration of potassium chloride (in most cases I = 50 mM). For Henry's function $F(\kappa a)$ we used the approximate mathematical expression introduced by Swan and Furst[8]:

$$F(\kappa a) = \frac{16 + 18\kappa a + 3(\kappa a)^2}{16 + 18\kappa a + 2(\kappa a)^2}. \qquad \text{Eq. 14}$$

The values determined for the ionic strength and Henry's function for each vesicle system are used to translate the measured electrophoretic mobility into the desired ζ-potential.

The overall workflow to determine the ζ-potential is summarised in **Scheme S1**.

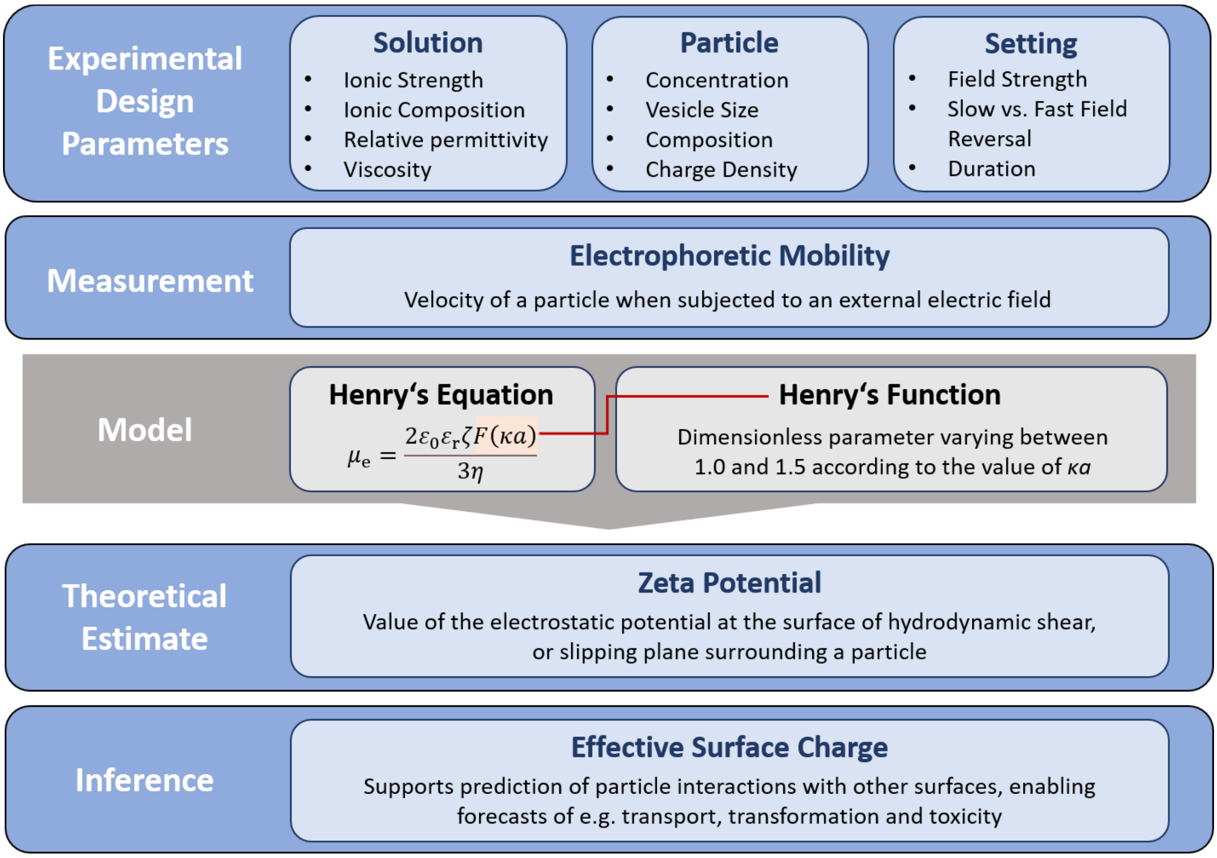

Scheme S1 - Relationships between the concepts and parameters involved with electrophoretic mobility measurements and ζ-potential estimates. Scheme adapted from Lowry et al.[9]

Calculation

The ζ-potentials determined from the measured electrophoretic mobilities are summarised in **Table S4,** for liposomes and proteoliposomes with the lipid composition of DOPC:DOPE:CL 8:1:1.

The thickness of the electric double layer (κ^{-1}) is the same for most of the performed measurements due to the constant measurement conditions for pH, ionic strength and ionic components inside the dispersant. Hence, only the change in vesicle radius (*a*) affects Henry's function. In most cases, the size of our vesicle systems is very similar, therefore the variations in F(*κa*) are minor. The main contribution to the changes in the presented ζ-potential values therefore derives from the different electrophoretic mobilities of the measured vesicle species.

Table S4 - Example of the transition from electrophoretic mobility to ζ-potential for liposomes and PLs with DOPC:DOPE:CL 8:1:1 lipid composition.

Sample	μ_e in µmcm/Vs	*a* in nm	κ in m^{-1}	κa	F(κa)	ζ in mV
Liposomes	-2.128	60.0	7.41 x 10^8	44.4	1.414	-28.8
PLs	-1.036	61.8	7.41 x 10^8	45.8	1.417	-14.0

μ_e = electrophoretic mobility, *a* = vesicle radius, κ = inverse Debye length, F(*κa*) = Henry's function, ζ = zeta potential

4 Derivations for calculated values

4.1 Average number of cardiolipins and R-CI per proteoliposome

Average number of R-CI per PL

To determine the average number of R-CI in PLs, the combination of the total protein concentration ($c_{\text{R-CI}}$; NADH:APAD$^+$ assay) and number of vesicles is needed. The total protein content is required to calculate the number of R-CI molecules inside the measurement cell (sample volume V = 0.06 mL); dividing this number by the measured number of vesicles yields the average number of enzymes per PL.

For example, the value of 34.6 R-CI units in PLs with DOPC:DOPE:CL 8:1:1 prepared under a protein-to-lipid ratio of 1:25 was obtained using $N_{\text{ves}} = 3.4 \pm 0.5 \text{ x } 10^{10}$ and $c_{\text{R-CI}} = 198.6 \pm 16.3 \frac{\mu\text{g}}{\text{mL}}$. The total protein concentration was reduced by a factor of 100 in line with the MADLS measurement settings. The total amount of R-CI molecules in the solution is then given by:

$$N_{\text{R-CI}} = \frac{c_{\text{R-CI}} \cdot V}{M_{\text{R-CI}}} \cdot N_{\text{A}} = 7.11 \text{ x } 10^{11} \,, \qquad \text{Eq. 15}$$

where $M_{\text{R-CI}} = 980 \text{ kDa}$.[10] Dividing $N_{\text{R-CI}}$ with the measured number of vesicles from MADLS gives the average number of R-CI units per PL of 34.6.

Average number of CLs per PL

Based on the measured total lipid concentration and the applied lipid mixture, we can use the number of vesicles from the MADLS to determine the amount of each lipid type in the lipid bilayer.

For example, the number of $1.84 \text{ x } 10^4$ CLs for the 10 wt% lipid mixture is based on the following values, accounting for the dilution (200-fold) used for the MADLS measurement and the sample volume inside the cuvette:

- $N_{\text{ves}}(\text{PLs}) = 1.58 \text{ x } 10^{10}$
- $c_{\text{lipid}}(\text{PLs}) = 7.60 \frac{\text{mg}}{\text{mL}}$
- $M_{\text{DOPC}} = 786.11 \frac{\text{g}}{\text{mol}}$; $M_{\text{DOPE}} = 744.03 \frac{\text{g}}{\text{mol}}$; $M_{\text{CL}} = 1501.96 \frac{\text{g}}{\text{mol}}$
- $c_{\text{DOPC}} = 0.032 \frac{\text{mg}}{\text{mL}}$; $c_{\text{DOPE}} = 0.004 \frac{\text{mg}}{\text{mL}}$; $c_{\text{CL}} = 0.004 \frac{\text{mg}}{\text{mL}}$

With the adjusted concentrations, N_{tot} for each lipid component can then be calculated (**Table S5**).

Table S5 - Summary of the derived number of lipids (N_{tot}) per PL, grouped by the phospholipid present inside the lipid bilayer structure

	DOPC	DOPE	CL
N_{lipid}	2.42 x 10^{15}	3.20 x 10^{14}	1.58 x 10^{14}
N		2.90 x 10^{15}	
N_{tot}		1.84 x 10^{5}	
N_{tot} per lipid	1.47 x 10^{5}	1.84 x 10^{4}	1.84 x 10^{4}

4.2 Contributions to Δζ and average number of 'associated' CLs to reconstituted R-CI

The calculated change in ζ-potential is affected by interconnected parameters such as protein-lipid interactions, the total number of reconstituted R-CI as well as number of outward-facing hydrophilic domains of the protein. Moreover, at higher protein:lipid ratios, protein-protein interactions can contribute to the measured effective surface charge. By comparing changes in Δζ with the changes for outward-facing R-CI (Table S6), an estimate of the contribution of protein-lipid interactions and the amount of outward-facing R-CI to Δζ can be obtained.

Table S6. Calculation of the impact of outward-facing R-CI domains and protein-lipid interactions (especially CL) on the measured change in ζ-potential defined as $\Delta\zeta = \zeta(\text{PLs}) - \zeta(\text{liposomes})$. Data sets are taken from Figure 2E and Table 1 (variable lipid compositions with fixed protein:lipid ratio of 1:25).

System	Δζ in mV	$N_{R\text{-}CI}$ (outward)	Δζ relative to DOPC		$N_{R\text{-}CI}$ change relative to DOPC		Impact of R-CI	Impact of CL 'associated'
			in mV(1)	in %(2)	(3)	in %(4)	in %(5)	in %(6)
DOPC (reference)	1.7	3.7	n/a	n/a	n/a	n/a	n/a	n/a
DOPC:DOPE	2.9	6.3	1.2	70.6	2.6	70.3	99.5	0.5
DOPC:CL	15.3	28.1	13.6	800.0	29.9	659.5	82.4	17.6
DOPC:DOPE:CL	15.4	25.2	13.7	805.9	25.4	581.1	**72.1**	**27.9**

(1) Δζ (of given lipid mixture) minus Δζ(DOPC). (2) Result from (1) divided by Δζ(DOPC) times 100. (3) $N_{R\text{-}CI}$ (of given lipid mixture) minus $N_{R\text{-}CI}$ (DOPC). (4) Result from (3) divided by $N_{R\text{-}CI}$ (DOPC). (5) Result from (4) divided by (2). (6) 100% minus from result in (5).

The comparisons indicate that the change in the effective surface charge from PLs containing only DOPC and DOPE relative to the 'optimised' composition is dominated by the increased amount of outward-facing R-CI (~72% contribution). The 72% estimate from Table S6 can be used to determine how many CL molecules are 'associated' per reconstituted R-CI (Table S7). See main text for a discussion of the meaning of 'associated' CL with R-CI.

Table S7. Calculation of the average number of associated CL molecules per reconstituted R-CI for the 'optimised' lipid mixture. The table combines two data sets for DOPC:DOPE:CL presented in Figure 2E/Table 1.

Parameter	Sample	
	'1:25' (Figure 2E)	'DOPC:DOPE:CL' (Figure 2E)
**CL per R-CI**	612 ± 70	593 ± 80
**Δζ in mV**	11.6 ± 1.1	15.4 ± 1.5
**Δζ in %***	39.3 ± 4.3	50.6 ± 5.0
**Adjusted Δζ in %****	11.0 ± 2.4	14.1 ± 2.8
**Adjusted 'associated' CL**	67 ± 16	84 ± 20

*The %-value describes the change in ζ-potential in comparison to the effective surface charge of the initial liposomes (black bars in Figure 2E). **The %-change in row 4 was obtained by reducing the value from row 3 by the percentage values presented in Table S6 (27.9% for data columns 2 and 3, hence 'adjusted Δζ'). Row 5 was obtained by multiplying the values in row 1 with those in row 4 ÷ 100.

4.3 Influence of AOX and CL content on R-CI activity

Table S8 – Comparison of the catalytic activity of reconstituted R-CI with increasing CL content for two distinct AOX contents (1 and 10 µg/mL).

CL content in wt%	AOX 10 µg/mL		AOX 1 µg/mL	
	activity in µmol/min per mg	(rel.) activity in %	activity in µmol/min per mg	(rel.) activity in %
**0**	4.0 ± 1.2	24.1 ± 6.0	3.3 ± 1.0	23.5 ± 5.5
**2.5**	6.7 ± 0.2	40.4 ± 3.5	5.6 ± 0.3	40.0 ± 4.0
**5.0**	10.1 ± 0.8	60.8 ± 4.0	8.5 ± 1.0	60.4 ± 5.0
**7.5**	12.6 ± 1.3	75.9 ± 5.5	11.0 ± 1.2	77.8 ± 5.2
**10.0**	16.6 ± 1.3	100 ± 4.5	14.1 ± 1.3	100 ± 4.5

5 Supplementary Figures

5.1 Intensity-weighted size distributions for liposomes and PLs before and after performing ELS measurements

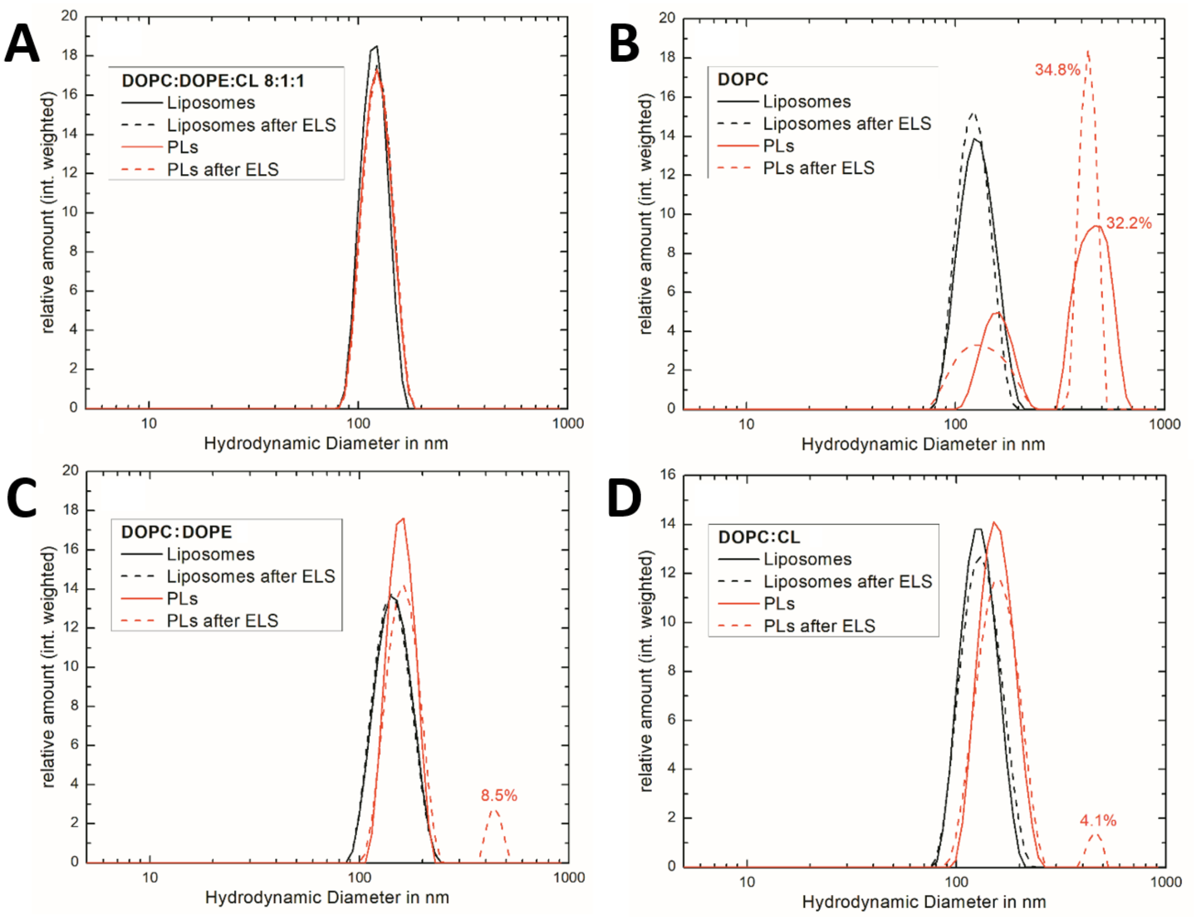

Figure S1 - Intensity-weighted size distributions for liposomes and PLs before and after performing ELS measurements. The results are separated based on lipid mixtures: **A –** optimised system with DOPC:DOPE:CL 8:1:1, **B –** DOPC, **C –** DOPC:DOPE, **D –** DOPC:CL.

5.2 Vesicle size distributions with increasing CL content

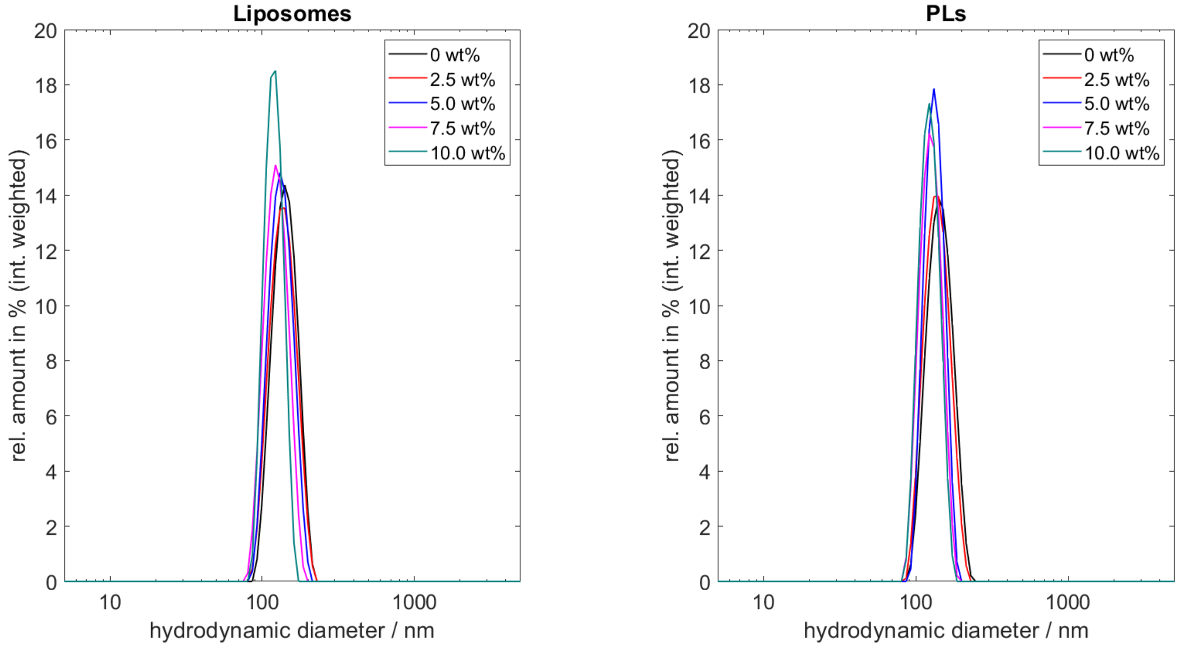

Figure S2 – MADLS vesicle size distributions for liposomes and PLs with increasing CL content.

5.3 Lipid concentration results

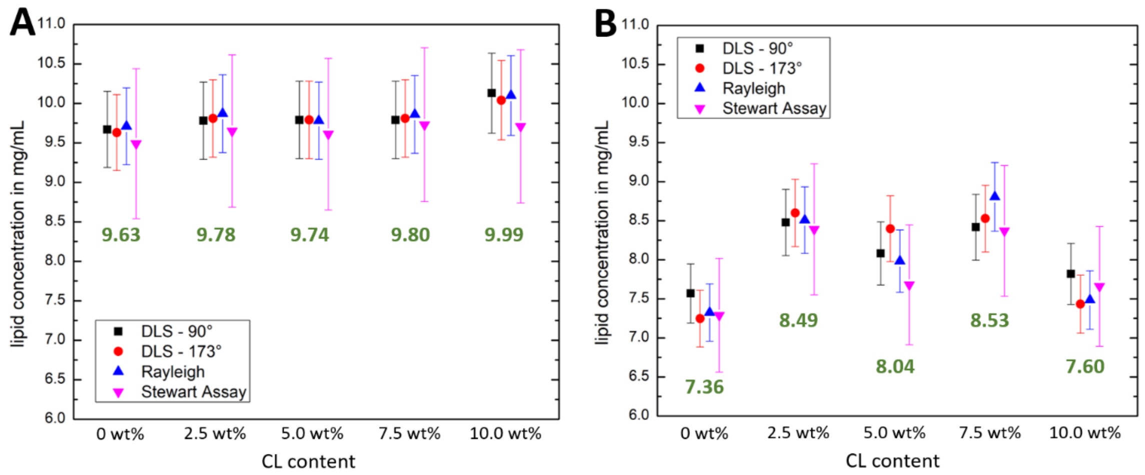

Figure S3 – Measured lipid concentrations for (A) liposomes (extrusion I samples) and (B) PLs. The two DLS detection angles used are plotted in combination with the results from the spectrofluorometric analysis as well as the results from the Stewart assay. The green numbers indicate the mean value based on all data points per lipid composition.

5.4 Aggregation of AOX and influence on liposomes

Before carrying out ζ-potential measurements, the influence of AOX on liposomes and PL size was investigated. Below an AOX concentration of 1.0 µg/mL (0.67:1 AOX to R-CI mass ratio), no significant changes in the size distributions are visible. At higher values, the size distribution shifts to a larger hydrodynamic diameter, indicating surface association of AOX on the lipid bilayer (see **Figure S4**). AOX alone at 10 µg/mL in aqueous buffer solution results in one peak at larger diameters, indicating

the presence of aggregated AOX (black traces). These aggregates are not present in the liposomes and PL vesicle systems, meaning that all the AOX is assembled on the surface.

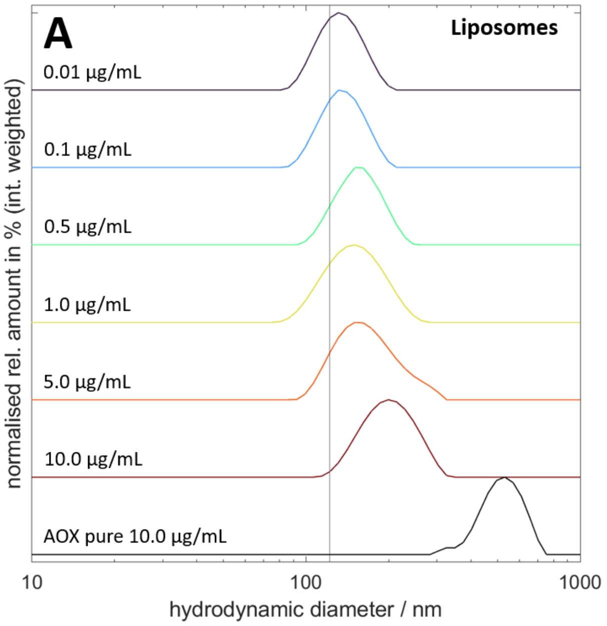

Figure S4 - Normalised number-weighted size distributions for liposomes (**A**) and PLs (**B**) with different amounts of AOX. The black trace presents a reference measurement of AOX alone (10 µg/mL) dissolved in reconstitution buffer.

For the same set of liposomes and PLs, the change in the mean hydrodynamic diameter and ζ-potential are shown in **Figure S5**.

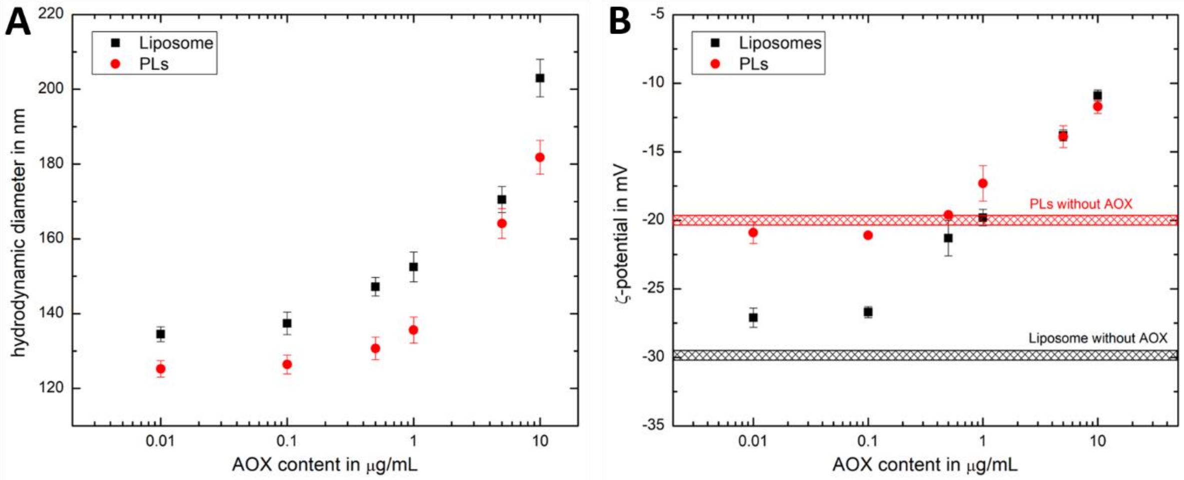

Figure S5 - Change in mean hydrodynamic diameter (**A**) and ζ-potential (**B**) with an increasing amount of AOX for liposomes and PLs with optimised composition (DOPC:DOPE:CL 8:1:1). The ζ-potential values for liposomes and PLs without AOX are included as a black and red bar, respectively. The data relate Figure S4. Note that the red data points in B are equivalent to the black data points in Figure 4Ai in the main text, except that they were obtained on a different batch of PLs (showing some variation in the data).

5.5 Checking the integrity of PLs after proton pumping measurements with ELS

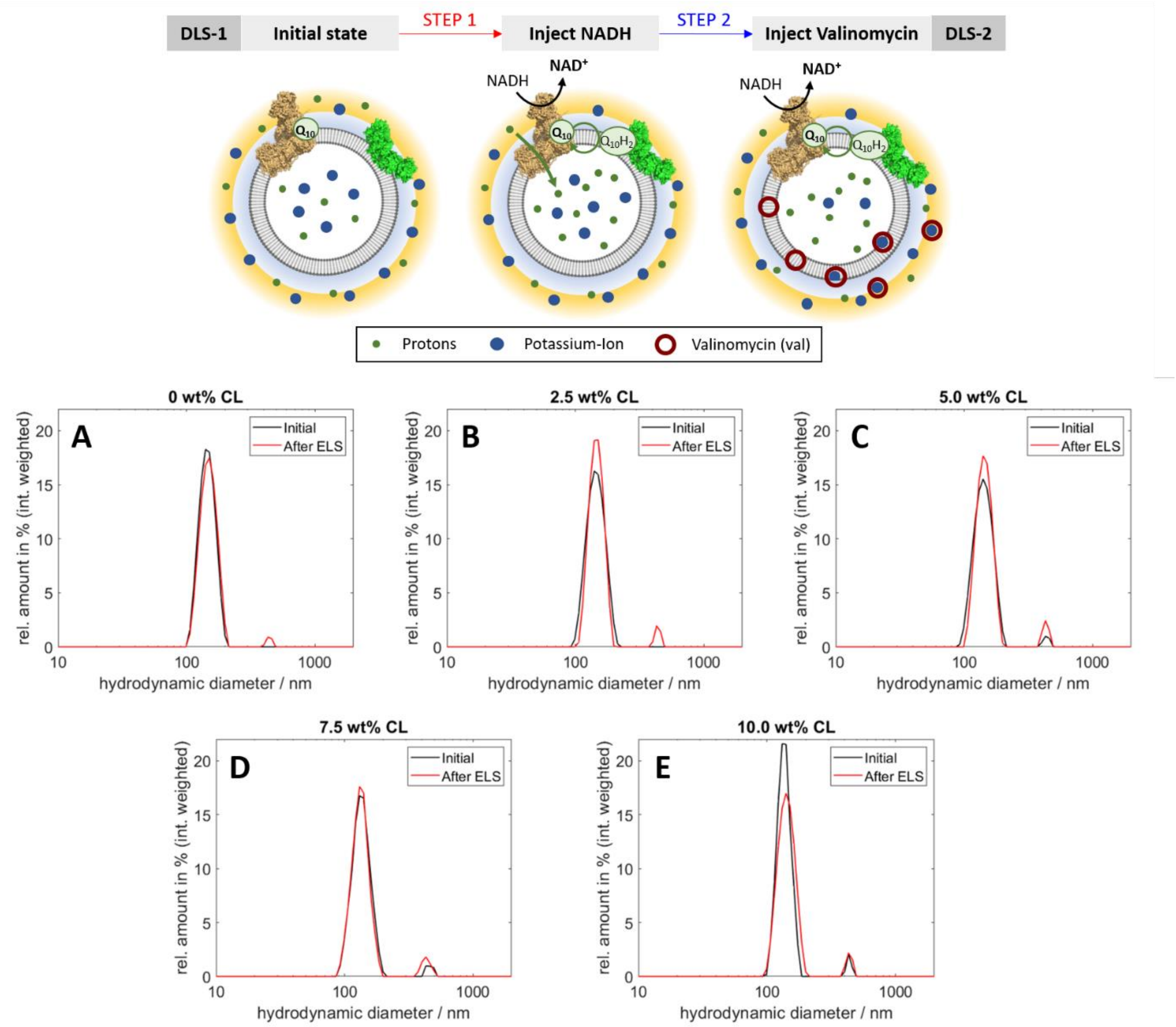

Figure S6 – **Top:** Schematic representation of the routine used to study proton pumping via ELS as well as DLS measurements to check the integrity of the vesicles. **Bottom:** Overlay of the MADLS size distributions for AOX-PLs before and after the ELS measurements to study proton pumping across the lipid bilayer. The lipid bilayers contained (**A**) 0 wt% CL, (**B**) 2.5 wt% CL, (**C**) 5 wt% CL, (**D**) 7.5 wt% CL and (**E**) 10 wt% CL.

5.6 Change in ζ-potential with addition of NADH and valinomycin

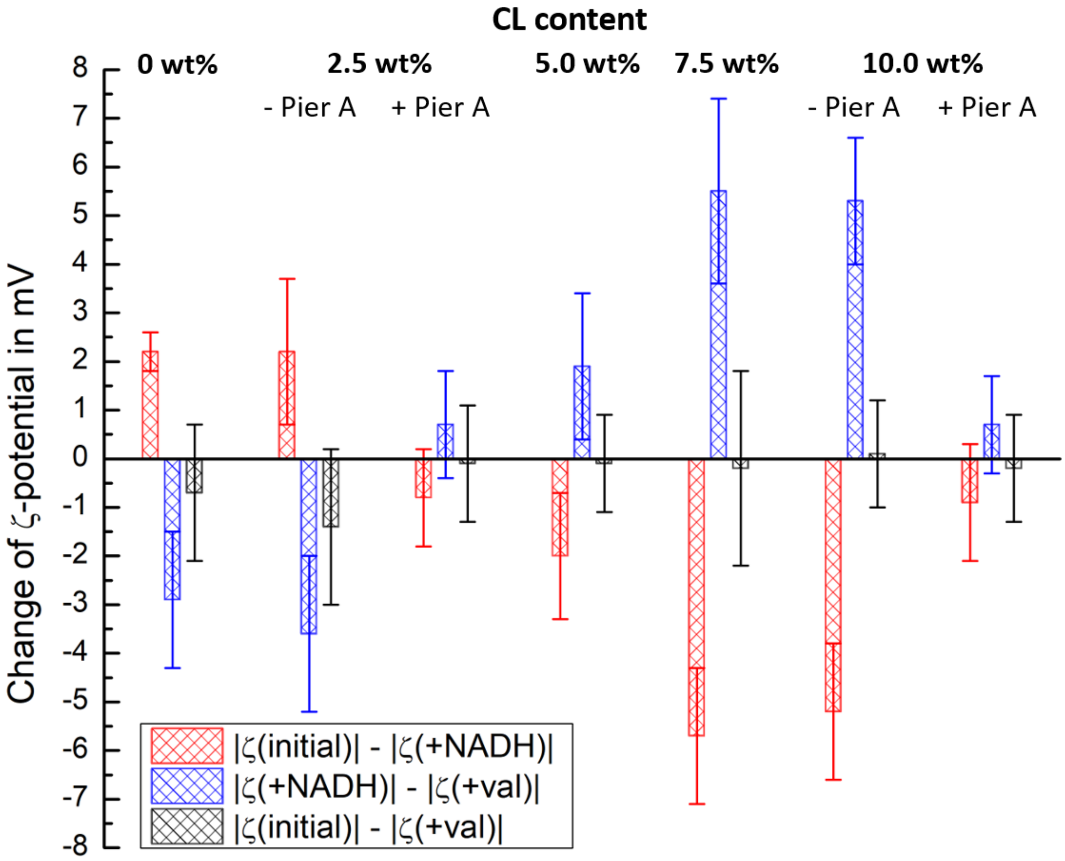

Figure S7 – Difference in ζ-potential after the addition of NADH (black bars), valinomycin (red bars) and the overall change (blue bars) with increasing CL content inside the lipid bilayer.

Figure S7 is an alternative representation of the data in Figure 4Bi, i.e. the red/blue arrows correspond to the red/blue bars in **Figures 4Bi** and **S7**, respectively. Control experiments with piericidin (at 2.5 wt% and 10 wt% CL) show that there are no significant ζ-potential changes in the presence of this R-CI inhibitor (the black/red/blue bars are all zero within error).

5.7 Correlation between ζ-potential and biochemical parameters

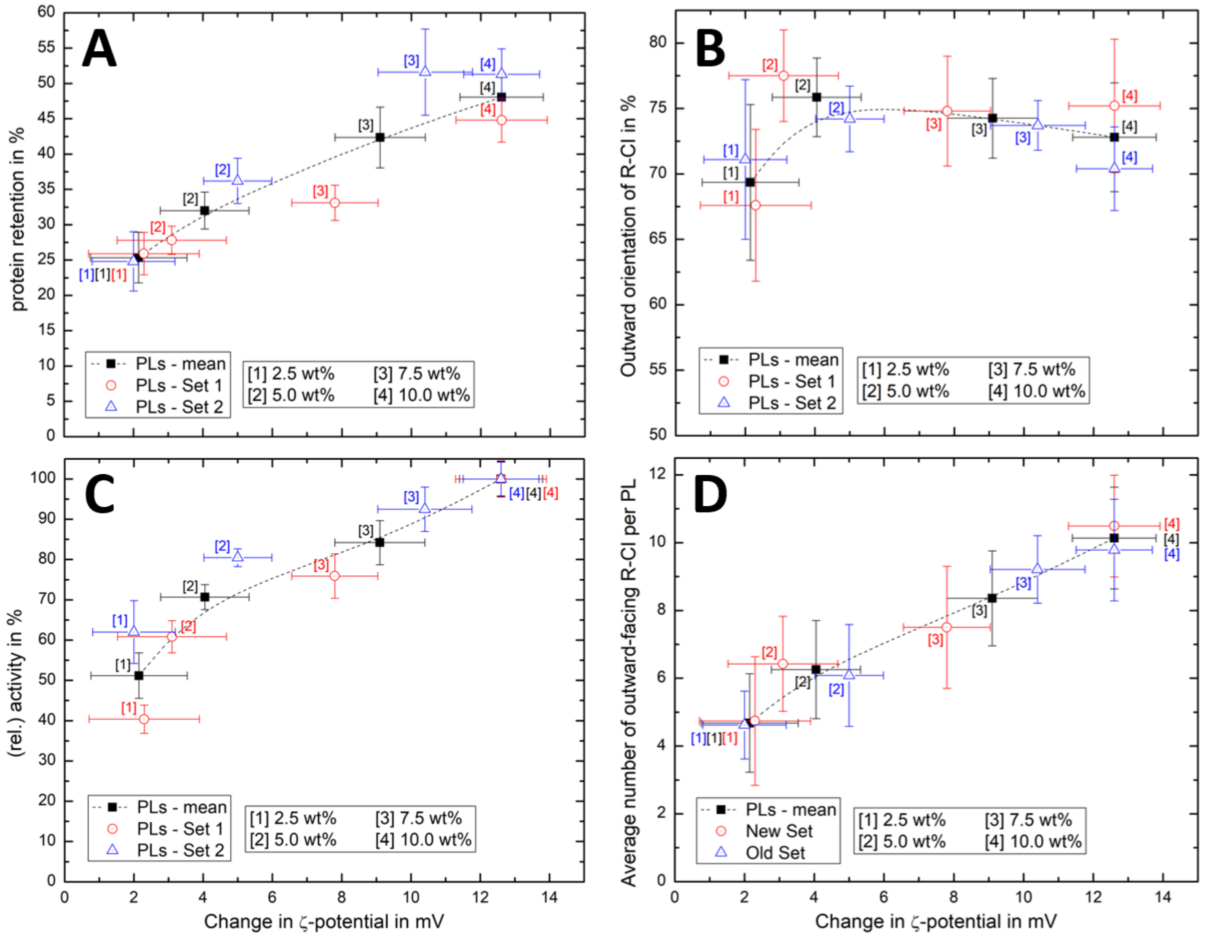

Figure S8 - Change in ζ-potential between liposomes and PLs containing 2.5-10 wt% CL plotted against **(A)** protein retention, **(B)** outward orientation, **(C)** catalytical activity (as relative value using the 10 wt% CL mixture as reference point) and **(D)** average amount of outward-facing R-CI. The mean values for the two measured PL sets (with protein:lipid ratio 1:50) are plotted as well as the two data sets individually.

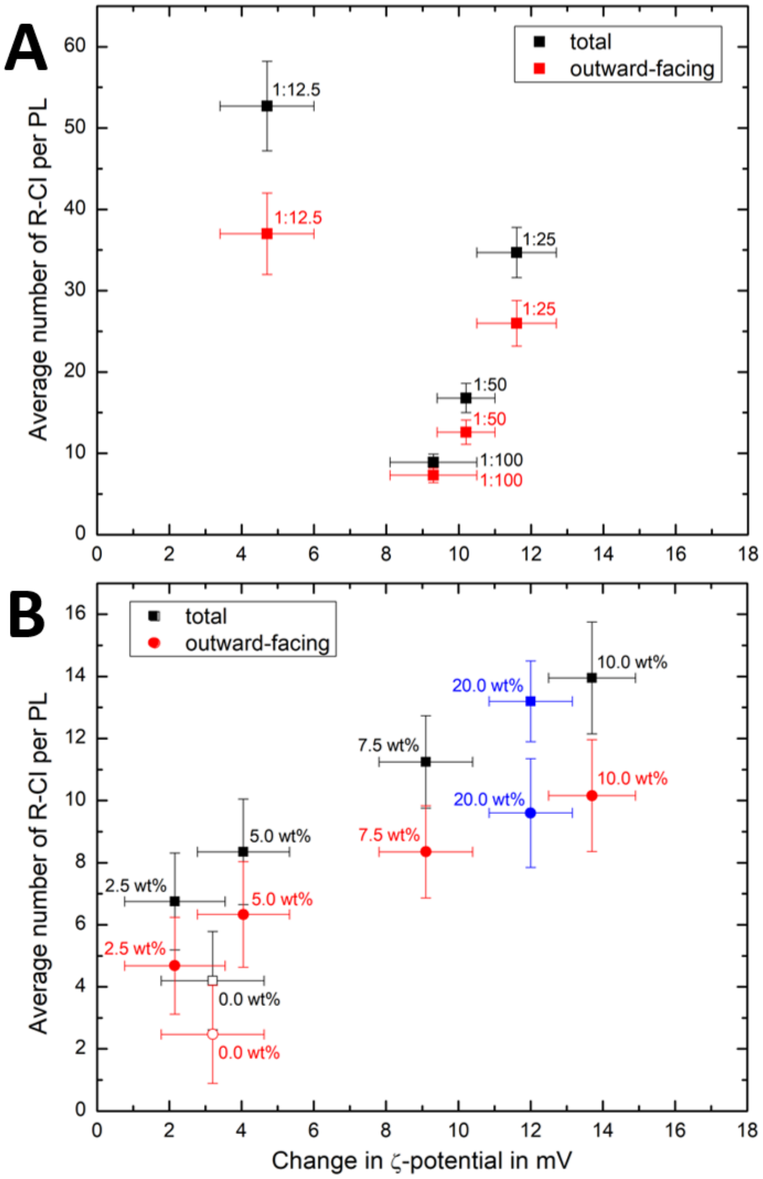

Figure S9 - Change in ζ-potential between liposomes and PLs with (**A)** an increase in protein:lipid ratio (fixed 10 wt% CL) and (**B)** increase CL content (fixed 1:50 protein:lipid ratio) against the total average number (black squares) and outward-facing (red circles) amount of reconstituted R-CI per PL. For A, data points correlate with results shown in Figure 2B and Table 1, whereas panel B presents the mean values for two measured PLs sets are shown (see Figure S8 for individual data sets); data points linked to 0 wt% CI are shown as hollow symbols. The primary data for the 20 wt% CL sample (mean value from two separate PL batches) can be found in Table S9.

The primary data set for the presented 20 wt% CL lipid mixture in Figures 5Bi and Bii are summarised in **Table S9**.

Table S9 - Overview of all determined physical and biochemical parameters for PL batches containing 20 wt% CL in the lipid mixture (protein:lipid ratio 1:50). Mean values with standard deviations from two sets of PLs are shown

**Parameter**	**20 wt% CL**
d_H (liposome) in nm	128.0 ± 2.0
ζ(liposome) in mV	-38.0 ± 1.0
N_{ves}(liposome)	1.56 ± 0.21 x 10^{11}
d_H (PLs) in nm	129.3 ± 2.2
ζ(PLs) in mV	-26.0 ± 1.2
N_{ves} (PLs)	5.68 ± 0.35 x 10^{10}
retention in %	47.5 ± 4.1
orientation in %	72.7 ± 5.5
activity in µmol/min per mg	17.5 ± 1.0
$N_{R\text{-}CI}$ per PL (average)	13.2 ± 1.3

d_H = hydrodynamic diameter; ζ = zeta potential; N_{ves} = number of vesicles

5.8 Electrostatic charge on the surface of R-CI

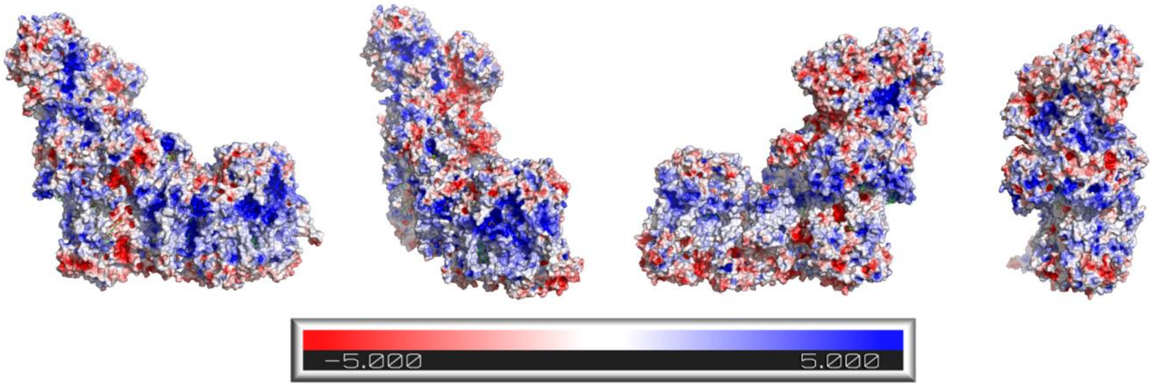

Figure S10 – Surface representation (calculated with PyMOL, APBS plugin) of bovine R-CI (PDB 7QSD).

6 References

1 O. Biner, J. G. Fedor, Z. Yin and J. Hirst, *ACS Synth. Biol.*, 2020, **9**, 1450–1459.

2 M. Naiim, A. Boualem, C. Ferre, M. Jabloun, A. Jalocha and P. Ravier, *Soft Matter*, 2015, **11**, 28–32.

3 T. Wriedt, in *The Mie Theory: Basics and Applications*, 2012, pp. 53–71.

4 Malvern Panalytical, *Particle Concentration Measurements on the Zetasizer Ultra – How It Works*, 2019.

5 G. Di Prima, F. Librizzi and R. Carrotta, *Membranes.*, 2020, **10**, 1–14.

6 S. Bhattacharjee, *J. Control. Release*, 2016, **235**, 337–351.

7 B. Petrillo and C. Moitzi, *Faster, More Sensitive Zeta-Potential Measurements with cmPALS and the Litesizer 500.*, 2016.

8 J. W. Swan and E. M. Furst, *J. Colloid Interface Sci.*, 2012, **388**, 92–94.

9 G. V. Lowry, R. J. Hill, S. Harper, A. F. Rawle, C. O. Hendren, F. Klaessig, U. Nobbmann, P. Sayre and J. Rumble, *Environ. Sci. Nano*, 2016, **3**, 953–965.

10 R. G. Efremov, R. Baradaran and L. A. Sazanov, *Nature*, 2010, **465**, 441–445.
